# A single-cell spiking model for the origin of grid-cell patterns

**DOI:** 10.1371/journal.pcbi.1005782

**Published:** 2017-10-02

**Authors:** Tiziano D’Albis, Richard Kempter

**Affiliations:** 1 Institute for Theoretical Biology, Department of Biology, Humboldt-Universität zu Berlin, Berlin, Germany; 2 Bernstein Center for Computational Neuroscience Berlin, Berlin, Germany; 3 Einstein Center for Neurosciences Berlin, Berlin, Germany; University of Hertfordshire, UNITED STATES

## Abstract

Spatial cognition in mammals is thought to rely on the activity of grid cells in the entorhinal cortex, yet the fundamental principles underlying the origin of grid-cell firing are still debated. Grid-like patterns could emerge via Hebbian learning and neuronal adaptation, but current computational models remained too abstract to allow direct confrontation with experimental data. Here, we propose a single-cell spiking model that generates grid firing fields via spike-rate adaptation and spike-timing dependent plasticity. Through rigorous mathematical analysis applicable in the linear limit, we quantitatively predict the requirements for grid-pattern formation, and we establish a direct link to classical pattern-forming systems of the Turing type. Our study lays the groundwork for biophysically-realistic models of grid-cell activity.

## Author summary

When an animal explores an environment, grid cells activate at multiple spatial locations that form a strikingly-regular triangular pattern. Grid cells are believed to support high-level cognitive functions such as navigation and spatial memory, yet the origin of their activity remains unclear. Here we focus on the hypothesis that grid patterns emerge from a competition between persistent excitation by spatially-selective inputs and the reluctance of a neuron to fire for long stretches of time. Using a computational model, we generate grid-like activity by only spatially-irregular inputs, Hebbian synaptic plasticity, and neuronal adaptation. We study how the geometry of the output patterns depends on the spatial tuning of the inputs and the adaptation properties of single cells. The present work sheds light on the origin of grid-cell firing and makes specific predictions that could be tested experimentally.

## Introduction

Grid cells are neurons of the medial entorhinal cortex (mEC) tuned to the position of the animal in the environment [1, 2]. Unlike place cells, which typically fire in a single spatial location [3, 4], grid cells have multiple receptive fields that form a strikingly-regular triangular pattern in space. Since their discovery, grid cells have been the object of a great number of experimental and theoretical studies, and they are thought to support high-level cognitive functions such as self-location [e.g. 5, 6], spatial navigation [e.g. 7–9], and spatial memory [10, 11]. Nevertheless, to date, the mechanisms underlying the formation of grid spatial patterns are yet to be understood [12, 13].

The attractor-network theory proposes that grid fields could arise from a path-integrating process, where bumps of neural activity are displaced across a low-dimensional continuous attractor by self-motion cues [14–21]. The idea that self-motion inputs could drive spatial firing is motivated by the fact that mammals can use path integration for navigation [22], that speed and head-direction signals have been recorded within the mEC [23, 24], and that, in the rat [1, 25] but not in the mouse [26, 27], grid firing fields tend to persist in darkness. However, grid-cell activity may rely also on non-visual sensory inputs—such as olfactory or tactile cues—even in complete darkness [28]. Additionally, the attractor theory alone cannot explain how grid fields are anchored to the physical space, and how the properties of the grid patterns relate to the geometry of the enclosure [29–31].

A different explanation for the formation of grid-cell activity is given by the so-called oscillatory-interference models [32–36]. In those models, periodic spatial patterns are generated by the interference between multiple oscillators whose frequencies are controlled by the velocity of the animal. Speed-modulated rhythmic activity is indeed prominent throughout the hippocampal formation in rodents and primates [37–40], particularly within the theta frequency band (4-12 Hz). Additionally, reduced theta rhythmicity disrupts grid-cell firing [41, 42], and grid-cell phase precession [43] is intrinsically generated by interference models; but see [44]. Despite their theoretical appeal, however, these models cannot explain grid-cell activity in the absence of continuous theta oscillations in the bat [45], and they are inconsistent with the grid-cell membrane-potential dynamics as measured intracellularly [46, 47]; see [48] for a hybrid oscillatory-attractor model.

Here we focus on the idea that grid-cell activity does not originate from self-motion cues, but rather from a learning process driven by external sensory inputs. In particular, it was proposed that grid patterns could arise from a competition between persistent excitation by spatially-selective inputs and the reluctance of a neuron to fire for long stretches of time [49–53]. In this case, Hebbian plasticity at the input synapses could imprint a periodic pattern in the output activity of a single neuron. Spatially-selective inputs, i.e., inputs with significant spatial information, are indeed abundant within the mEC [54–56] and its afferent structures [57–61] And spike-rate adaptation, which is ubiquitous in the brain [62], could hinder neuronal firing in response to persistent excitation.

Kropff and Treves [49] explored this hypothesis by means of a computational model; see also [63–67] and Sec Related models for similar works. The emergence of grid-like patterns was demonstrated with theoretical arguments and with numerical simulations of a rate-based network. However, because of a relatively abstract level of description, the outcomes of the model could not be easily confronted with experimental data. Specifically, the simulations included a network-level normalization mechanism that constrained the mean and the sparseness of the output activity, and it remained unsettled whether grid patterns could emerge in a single-cell scenario. Additionally, the synaptic weights did not obey Dale’s law. And the robustness of the model was not tested against shot noise due to stochastic spiking. Finally, the link between the numerical simulations and the underlying mathematical theory remained rather loose.

To overcome these issues, we propose here a single-cell spiking model based on similar principles as the model by Kropff and Treves [49], but that is, on the one hand, more biologically realistic, and on the other hand, better suited for mathematical treatment. Importantly, we show that grid patterns can emerge from a single-cell feed-forward mechanism needless of any network-level interaction (although recurrent dynamics may be still required to explain the coherent alignment of grid patterns [1]). To increase biological plausibility, we consider a stochastic spiking neuron model, and we constrain the synaptic weights to non-negative values (Dale’s law). Finally, by studying the model analytically, we quantitatively predict the requirements for grid-pattern formation, and we establish a direct link to classical pattern-forming systems via the Turing instability [68].

## Results

### Model of neural activity

We consider a single cell that receives synaptic input from *N* spatially-tuned excitatory neurons. Input spike trains $S_{i}^{\text{in}}\left(t\right)≔\sum_{k}\;\delta \left(t-t_{i,k}^{\text{in}}\right)$ for *i* = 1, 2, …, *N* are generated by independent inhomogeneous Poisson processes with instantaneous rates $r_{i}^{\text{in}}\left(t\right)$ where *δ*(*t*) is the Dirac delta function, and $t_{i,k}^{\text{in}}$ is the timing of the *k*^th^ input spike at synapse *i*. Similarly, the output spike train $S^{\text{out}}\left(t\right)≔\sum_{k}\;\delta \left(t-t_{k}^{\text{out}}\right)$ is generated by an inhomogeneous Poisson process with instantaneous rate *r*^out^(*t*) where $t_{k}^{\text{out}}$ denotes the timing of the *k*^th^ output spike.

We assume that inputs are integrated linearly at the output, and that the output neuron is equipped with an intrinsic spike-rate adaptation mechanism, that is,

$$\begin{array}{c} r^{\text{out}}\left(t\right)≔r_{0}+\int_{0}^{\infty }\;d\tau \;\;K\left(\tau \right)\;\;\sum_{i=1}^{N}\;w_{i}S_{i}^{\text{in}}\left(t-\tau \right) \end{array}$$ (1)

where *r*_0_ is a baseline rate, *w*_*i*_ is the synaptic weight of input neuron *i*, and the function *K* is a temporal filter modeling the spike-rate adaptation dynamics. Note that the instantaneous output rate *r*^out^ depends only on the temporal history of the input spikes and that there is no reset mechanism after the emission of an output spike.

**Fig 1:**
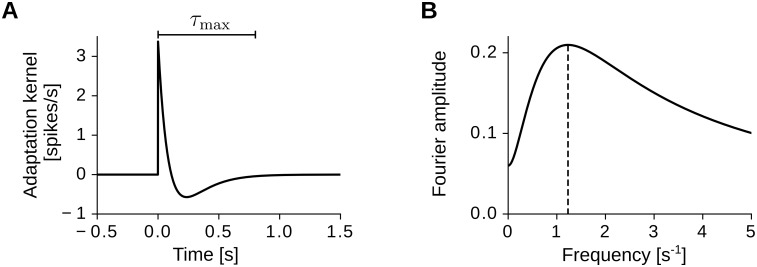
The temporal adaptation kernel *K*. (A) Impulse response of the filter (Eq 2). A positive peak with amplitude *K*(0) = 1/*τ*_S_ − *μ*/*τ*_L_ ≈ 3.4 spikes/s is followed by a slow negative response. Note that the kernel is small for *t* > *τ*_max_, i.e., |*K*(*t*)| < 0.01|*K*(0)| for *t* > *τ*_max_, with *τ*_max_ = 5*τ*_L_ = 0.8 s. (B) Frequency response of the filter. The dashed vertical line indicates the filter’s resonance frequency *k*_res_ = 1.23 s^−1^. Parameter values: *τ*_S_ = 0.1 s, *τ*_L_ = 0.16 s, *μ* = 1.06. The integral of the filter is 1 − *μ* = −0.06.

The impulse response of the adaptation kernel *K* is the sum of two exponential functions:

$$\begin{array}{c} K\left(t\right)≔\{\begin{array}{cc} \frac{1}{\tau_{\text{S}}}\exp\;(-\frac{t}{\tau_{\text{S}}})-\frac{\mu }{\tau_{\text{L}}}\exp\;(-\frac{t}{\tau_{\text{L}}}) & \text{for}\;t\geq 0\\ 0 & \text{for}\;t<0 \end{array} \end{array}$$ (2)

where *τ*_S_ and *τ*_L_ are the short and long filter time constants (0 < *τ*_S_ < *τ*_L_), and the parameter *μ* > 0 sets the filter integral $\int_{0}^{\infty }\;dt\;K\left(t\right)=1-\mu$ (Fig 1A). Intuitively, at the arrival of an input spike, the firing probability of the output neuron is first increased for a short time that is controlled by the time constant *τ*_S_, and then decreased for a longer time that is controlled by the time constant *τ*_L_. This second hyper-polarization dynamics effectively hinders the neuron to fire at high rates for long stretches of time, mimicking a spike-rate adaptation mechanism [69–71]. From a signal-processing perspective, the adaptation kernel *K* performs a temporal band-pass filtering of the input activity (Fig 1B), and the two time constants *τ*_S_ and *τ*_L_ control the resonance frequency *k*_res_ at which the filter response is maximal. Note that in Sec Pattern formation with after-spike potentials we study a variant of the present model where neuronal adaptation is obtained though after-spike hyperpolarizing potentials associated to the output activity of the neuron.

### Model of synaptic plasticity

We assume spike-timing dependent plasticity (STDP) at the input synapses [e.g. 72–76]. Input and output spikes trigger weight changes Δ*w*_*i*_ according to the following rule:

1. For each pair of a post-synaptic spike and a pre-synaptic spike at synapse *i*, we set
  $$\begin{array}{c} \Delta w_{i}=\eta W\left(\Delta t\right) \end{array}$$ (3)
2. For each pre-synaptic spike at synapse *i*, we set
  $$\begin{array}{c} \Delta w_{i}=\eta \left(\beta -\alpha w_{i}\right) \end{array}$$ (4)

where *η* ≪ 1 is a small learning rate, and the STDP learning window *W*(Δ*t*) sets the weight change as a function of the time difference Δ*t* ≔ *t*_pre_ − *t*_post_ between pre- and post-synaptic spikes. We consider a symmetric STDP learning window [77]

$$\begin{array}{c} W\left(\Delta t\right)≔\frac{W_{\text{tot}}}{2\tau_{\text{W}}}\exp(-|\Delta t|/\tau_{\text{W}}) \end{array}$$ (5)

where the time constant *τ*_W_ > 0 controls the maximal time lag at which plasticity occurs, and $W_{\text{tot}}=\int_{-\infty }^{\infty }\;dt\;W\left(t\right)$ is the integral of the learning window. The first part of the learning rule (Eq 3) is the classical Hebbian term whereas the second part (Eq 4) is a local normalization term that stabilizes the average synaptic strength $w_{\text{av}}=N^{-1}\sum_{i=1}^{N}w_{i}$ and prevents the individual weights to grow unbounded. This normalization term mimics local homoeostatic processes observed experimentally [78–80]; see also [81] for a review. The parameters *α* > 0 and *β* set, respectively, the rate of weight decay and the target average weight *w*_av_ (Sec Weight normalization). Importantly, the synaptic weights are constrained to non-negative values by imposing the hard bounds

$$\begin{array}{c} w_{i}\geq 0\;\;\forall i\;. \end{array}$$ (6)

### Model of input spatial tuning

We consider excitatory inputs with firing rates $r_{i}^{\text{in}}$ that are tuned to the spatial position of a virtual rat exploring a square arena of side-length *L*, i.e.,

$$\begin{array}{c} r_{i}^{\text{in}}\left(t\right)≔\Psi_{i}^{\text{in}}\left(x_{t}\right) \end{array}$$ (7)

where **x**_*t*_ is the position of the virtual rat at time *t*, and $\Psi_{i}^{\text{in}}$ is a spatial tuning curve. We characterize the spatial tuning curves $\Psi_{i}^{\text{in}}$ in two alternative scenarios:

1. *spatially-regular* inputs, i.e., each input has a single spatial receptive field;
2. *spatially-irregular* inputs, i.e., each input has multiple spatial receptive fields at random locations.

The first scenario, which is reminiscent of hippocampal place-cell activity [3, 82, 83], is easier to study analytically and cheaper to simulate numerically. The second scenario, which is reminiscent of parasubicular activity [57–61], is motivated by the anatomy of the entorhinal circuit (Sec Input spatial tuning and the origin of grid-cell patterns). In both cases, we consider circularly-symmetric receptive fields that cover the arena evenly. Indeed, place fields in open environments do not show systematic shape biases, and, in the absence of specific reward or goal locations, their centres are roughly homogeneously distributed [3, 57–61, 82, 83]. Note, however, that border-like inputs [84, 85]—which are not radially-symmetric—are present in the real system, but not explicitly modeled here. Finally, for simplicity, we assume periodic boundaries at the edges of the arena.

#### Spatially-regular inputs

In the case of *spatially-regular* inputs, we assume tuning curves of the form

$$\begin{array}{c} \Psi_{i}^{\text{in}}\left(x\right)≔G(|x-r_{i}\left|\right)\;\text{with}\;i=1,2,\ldots ,N \end{array}$$ (8)

where **r**_*i*_ is the receptive-field center of neuron *i* and G is a Gaussian function:

$$\begin{array}{c} G\left(r\right)≔\frac{L^{2}r_{\text{av}}}{2\pi \sigma^{2}}\exp\;(-\frac{r^{2}}{2\sigma^{2}})\;. \end{array}$$ (9)

The parameter *σ* > 0 sets the width of the receptive field, and *r*_av_ is the average firing rate in the environment. We assume that the input receptive-field centers **r**_*i*_ cover the entire arena evenly.

This input scenario is considered for the mathematical derivations in Sec Weight dynamics for spatially-regular inputs and for the numerical simulations in Secs Emergence of grid spatial patterns and Geometrical properties of the grid patterns.

#### Spatially-irregular inputs

In the case of *spatially-irregular* inputs, each tuning curve $\Psi_{i}^{\text{in}}$ is the sum of *M* > 1 Gaussian receptive fields with random amplitudes *A*_*ij*_ and random receptive-field centers **r**_*ij*_ with *i* = 1, 2, …, *N* and *j* = 1, 2, …, *M*, that is,

$$\begin{array}{c} \Psi_{i}^{\text{in}}\left(x\right)≔\frac{1}{\beta_{i}}\;\sum_{j=1}^{M}\;A_{ij}\;G(|x-r_{ij}|)\;. \end{array}$$ (10)

The scaling factors $\beta_{i}=\sum_{j=1}^{M}A_{ij}$ normalize the inputs $\Psi_{i}^{\text{in}}$ to the same average rate *r*_av_, and all the superimposed fields share the same field size *σ* (Eq 9). The field amplitudes *A*_*ij*_ are uniformly distributed in the range (0, 1), and the receptive-field centers **r**_*ij*_ are uniformly distributed in the environment.

This input scenario is considered for the mathematical derivations in Sec Eigenvalue spectrum for spatially-irregular inputs and for the numerical simulations in Sec Pattern formation with spatially-irregular inputs.

### Model of spatial exploration

The movement of the virtual rat follows a smooth random walk that satisfies the following three assumptions: (i) the movement speed *v* is constant in time; (ii) the random walk is isotropic and ergodic with respect to the auto-covariance; (iii) the virtual-rat trajectories are smooth within time stretches shorter than the time length *τ*_max_ = 5*τ*_L_ of the adaptation kernel *K* (Fig 1A). Note that assumption (i) is obviously not valid in general. However, because synaptic plasticity acts on a time scale that is much slower than behaviour, the relevant variable for pattern formation is the rat running speed averaged over long stretches of time (e.g. minutes), which can be considered approximately constant. We assume an average running speed of 25 cm/s, which is experimentally plausible [86]. Assumptions (ii) and (iii) hold by ignoring directional anisotropies deriving from the geometry of the environment, and by observing that experimental rat trajectories are approximately straight over short running distances (e.g, over distances shorter than 25 cm) [86].

**Fig 2:**
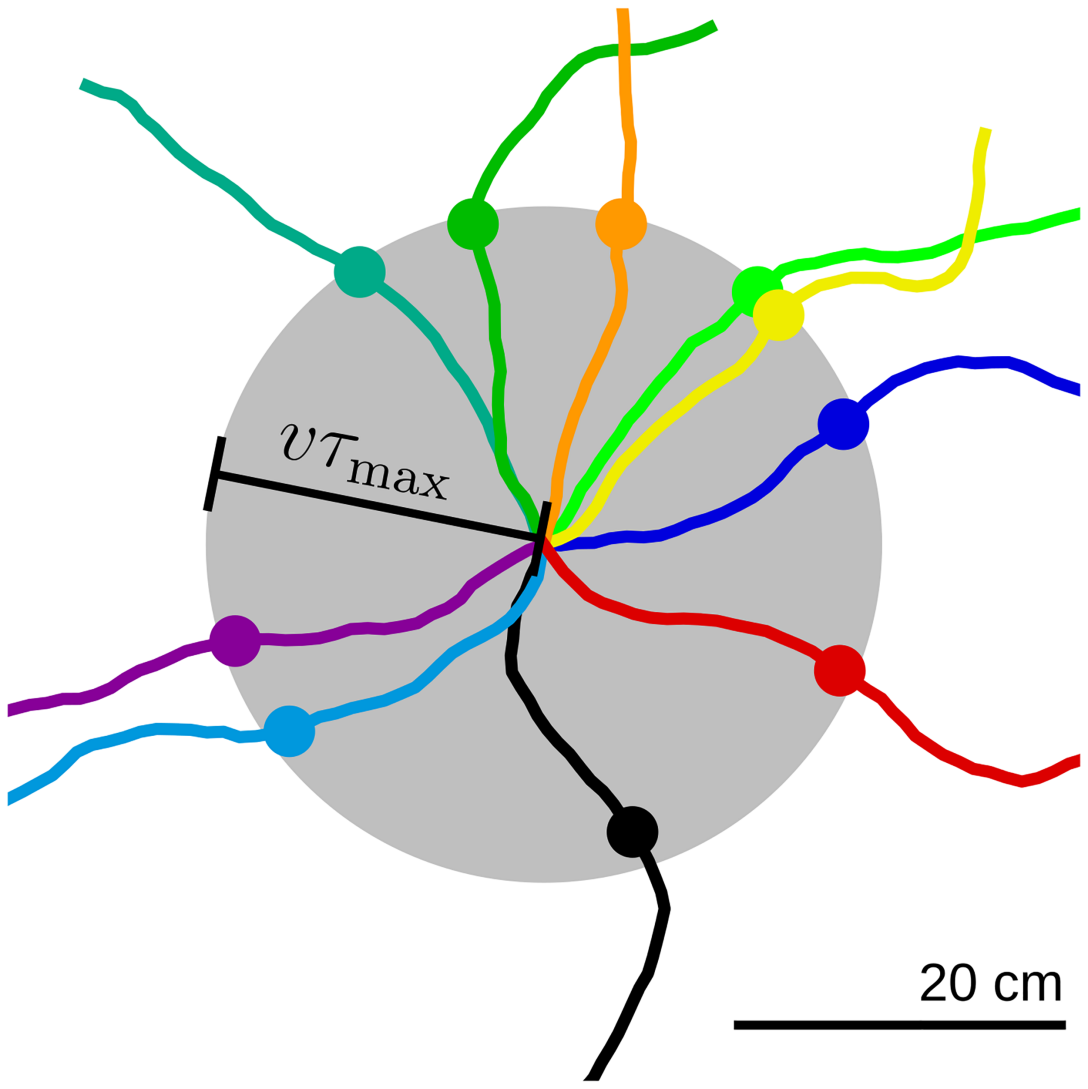
Example virtual-rat trajectories. Colored lines denote example virtual-rat trajectories obtained by integrating Eq 11 starting at the center of the gray disk. Filled dots indicate the position of the virtual rat at time *τ*_max_ = 5*τ*_L_ = 0.8 s. Note that the trajectories are smooth within time stretches shorter than *τ*_max_. Parameter values: *v* = 0.25 m/s, *θ*_*σ*_ = 0.7. The disk radius is *vτ*_max_ = 20 cm.

Mathematically, the two-dimensional virtual-rat trajectories **x**_*t*_ are sampled from the stochastic process

$$\begin{array}{c} \frac{dX_{t}}{dt}≔v\;\left[\cos\left(\theta_{t}\right),\sin\left(\theta_{t}\right)\right]\;\text{with}\;\theta_{t}=\sigma_{\theta }W_{t}\;, \end{array}$$ (11)

where the angle *θ*_*t*_ sets the direction of motion and $W_{t}$ is a standard Wiener process (Fig 2). The parameters *v* and *σ*_*θ*_ control the speed of motion and the tortuosity of the trajectory. Note that we also perform simulations with variable running speeds. In this case, the speed is sampled from an Ornstein-Uhlenbeck process with long-term mean $\bar{v}=v$.

### Mathematical results on grid-pattern formation

The grid-cell model presented above is studied both analytically and numerically. In this section, we obtain an equation for the average dynamics of the synaptic weights, and we derive the requirements for spatial pattern formation. In Sec Numerical results on grid-pattern formation we demonstrate the emergence of grid-like activity by simulating both the detailed spiking model and the averaged system. The analytical results presented here may be skipped by the less mathematically-inclined reader.

We study structure formation in the activity of an output cell by averaging the weight dynamics resulting from the stochastic activation of input and output neurons (Sec Model of neural activity) and the STDP learning rule (Sec Model of synaptic plasticity), while a virtual rat explores a two-dimensional enclosure and the inputs are spatially tuned (Secs Model of input spatial tuning and Model of spatial exploration). We take both ensemble averages across spike-train realizations and temporal averages within a time window of length *T*. The averaging time length *T* separates the time scale of neural activation (of the order of the width *τ*_W_ of the learning window *W*) from the time scale *τ*_str_ of structure formation, i.e., *τ*_W_ ≪ *T* ≪ *τ*_str_. Because *τ*_str_ is inversely proportional to the learning rate *η* (Eq 29), such averaging is always possible provided that the learning rate *η* is small enough. In other words, we assume that within a time *T*, the virtual rat has roughly explored the entire environment, but the synaptic weights did not change considerably. In this case, the dynamics of the synaptic weights *w*_*i*_ is approximated by a drift-diffusion process, where the deterministic drift term reads [74]

$$\begin{array}{c} \eta^{-1}\frac{d{\bar{w}}_{i}}{dt}=\left(\beta -\alpha {\bar{w}}_{i}\right)\bar{\langle S_{i}^{\text{in}}\left(t\right)\rangle }+\int_{-\infty }^{\infty }ds\;W\left(s\right)\bar{\langle S_{i}^{\text{in}}\left(t+s\right)S^{\text{out}}\left(t\right)}\rangle \; \end{array}$$ (12)

with ${\bar{w}}_{i}\geq 0$. The functions $S_{i}^{\text{in}}$ and *S*^out^ denote input and output spike trains (Sec Model of neural activity), the angular brackets denote ensemble averages over input and output spike trains, and the overbars denote temporal averages, i.e., $\bar{f}\left(t\right)≔T^{-1}\int_{t-T}^{t}\;ds\;f\left(s\right)$. Following Kempter et al. [74] we derive

$$\begin{array}{c} \bar{\langle S_{i}^{\text{in}}\left(t+s\right)S^{\text{out}}\left(t\right)}\rangle =\bar{\langle S_{i}^{\text{in}}\left(t+s\right)\rangle \langle S^{\text{out}}\left(t\right)\rangle }+{\bar{w}}_{i}\;\bar{\langle S_{i}^{\text{in}}\left(t\right)\rangle }K\left(-s\right)\;, \end{array}$$ (13)

where the ensemble averages read

$$\begin{array}{ccc} \langle S_{i}^{\text{in}}\left(t\right)\rangle & = & r_{i}^{\text{in}}\left(t\right) \end{array}$$ (14)

$$\begin{array}{ccc} \langle S^{\text{out}}\left(t\right)\rangle & = & \langle r^{\text{out}}\left(t\right)\rangle \;\overset{\left(1\right)}{=}\;r_{0}+\int_{0}^{\infty }\;d\tau \;\;K\left(\tau \right)\;\;\sum_{j=1}^{N}\;w_{j}r_{j}^{\text{in}}\left(t-\tau \right)\;. \end{array}$$ (15)

Finally, from Eqs 12–15 we obtain

$$\begin{array}{c} \eta^{-1}\frac{d}{dt}{\bar{w}}_{i}=\sum_{j=1}^{N}\;C_{ij}{\bar{w}}_{j}-a{\bar{w}}_{i}+b\;\text{with}\;{\bar{w}}_{i}\geq 0\; \end{array}$$ (16)

where we defined

$$\begin{array}{ccc} C_{ij} & ≔ & \int_{0}^{\infty }\;d\tau \;K\left(\tau \right)\int_{-\infty }^{\infty }ds\;W\left(s\right)\;\bar{r_{i}^{\text{in}}\left(t+s\right)r_{j}^{\text{in}}\left(t-\tau \right)} \end{array}$$ (17)

$$\begin{array}{ccc} a & ≔ & r_{\text{av}}\;[\alpha -\int_{-\infty }^{\infty }ds\;W\left(s\right)K\left(-s\right)] \end{array}$$ (18)

$$\begin{array}{ccc} b & ≔ & r_{\text{av}}\;(W_{\text{tot}}r_{0}+\beta )\;. \end{array}$$ (19)

Note that in deriving Eq 16 we approximated the temporal average of the input rates $\bar{r_{i}^{\text{in}}}$ with the spatial average *r*_av_ of the input tuning curves $\Psi_{i}^{\text{in}}$. This approximation holds with the assumption that in a time *T* the virtual rat roughly covers the entire space evenly.

By ignoring the non-linear weight constraints ${\bar{w}}_{i}\geq 0$, the average weight dynamics is described by a linear system with coupling terms *C*_*ij*_ (Eq 16). The coefficients *C*_*ij*_ are given by the temporal correlations of the input rates $r_{i}^{\text{in}}$ and $r_{j}^{\text{in}}$, filtered by the adaptation kernel *K* and the STDP learning window *W* (Eq 17).

To further simplify the calculations, we assume that the low-pass filtering introduced by the STDP learning window can be neglected for the purpose of studying pattern formation. In particular, we assume that the learning window *W* decays much faster than the changes in the input correlations $\bar{r_{i}^{\text{in}}\left(t+s\right)r_{j}^{\text{in}}\left(t-\tau \right)}$ (Eq 17), which holds for *τ*_W_ ≪ *σ*/*v*. In this case, we obtain

$$\begin{array}{c} C_{ij}\approx W_{\text{tot}}\int_{0}^{\infty }\;d\tau \;\;K\left(\tau \right)\;\;\bar{r_{i}^{\text{in}}\left(t\right)r_{j}^{\text{in}}\left(t-\tau \right)} \end{array}$$ (20)

where *W*_tot_ is the integral of the learning window (Eq 5).

Finally, by assuming smooth virtual-rat trajectories at constant speed *v*, the correlation matrix *C*_*ij*_ can be estimated solely from the input tuning curves $\Psi_{i}^{\text{in}}$ and the adaptation kernel *K* (Sec Input correlation for general inputs, Eq 47):

$$\begin{array}{c} C_{ij}\approx \frac{W_{\text{tot}}}{L^{2}}\int_{0}^{\infty }\;d\tau \;K\left(\tau \right)\;\oint_{|z|=\tau v}\;dz\;\;\Psi_{i}^{\text{in}}⋆\Psi_{j}^{\text{in}}{|}_{z} \end{array}$$ (21)

where *L*^2^ is the area explored by the virtual rat. In Eq 21, the matrix element *C*_*ij*_ is obtained by integrating the spatial cross-correlation of the input tuning curves $\Psi_{i}^{\text{in}}⋆\Psi_{j}^{\text{in}}$ over circles of radius *τv*, and by weighting each integral with the amplitude of the adaptation kernel *K* at time *τ*. Note that Eq 21 holds for generic spatial tuning curves $\Psi_{i}^{\text{in}}$.

#### Weight dynamics for spatially-regular inputs

To study the emergence of spatial patterns, we now consider the simplified scenario of spatially-regular inputs (Sec Spatially-regular inputs). That is, the input tuning curves $\Psi_{i}^{\text{in}}$ are circularly-symmetric Gaussian functions that cover the entire space evenly (Eqs 8 and 9). This input representation is particularly useful because it establishes a direct mapping between the neuron identity (the index *i*) and a position in physical space (the receptive-field center **r**_*i*_). Therefore, studying pattern formation in the activity of the output neuron is reduced to studying pattern formation in the space of the synaptic weights. Note, however, that such a simple input scenario is not necessary for the formation of grid-cell patterns in general, as shown in Sec Pattern formation with spatially-irregular inputs.

With spatially-regular inputs, the average weight dynamics in Eq 16 can be rewritten by labeling the synaptic weights according to the corresponding receptive-field centers **r**_*i*_:

$$\begin{array}{c} \eta^{-1}\frac{d}{dt}\bar{w}\left(r_{i}\right)=\sum_{i=1}^{N}\;C\left(r_{i},r_{j}\right)\bar{w}\left(r_{j}\right)-a\bar{w}\left(r_{i}\right)+b\; \end{array}$$ (22)

where $\bar{w}\left(r_{i}\right)={\bar{w}}_{i}$ and *C*(**r**_*i*_, **r**_*j*_) = *C_ij_*. Additionally, in the limit of a large number *N* ≫ 1 of input neurons and receptive fields that cover the environment with constant density *ρ* = *N*/*L*^2^, the sum in Eq 22 can be replaced by an integral over all the receptive-field centers **r**′:

$$\begin{array}{c} \eta^{-1}\frac{d}{dt}\bar{w}\left(r\right)=\rho \int dr^{\prime }\;C\left(r,r^{\prime }\right)\bar{w}\left(r^{\prime }\right)-a\bar{w}\left(r\right)+b \end{array}$$ (23)

where the correlation function *C*(**r**, **r**′) is the continuous extension of the correlation matrix *C_ij_* = *C*(**r**_*i*_, **r**_*j*_). Because the inputs are translation invariant (Eq 8), the correlation function *C* is also translation invariant, i.e., *C*(**r**, **r**′) = *C*(**r** − **r**′, **0**) = *C*(**r** − **r**′), where we omit the second argument **0** ≔ (0, 0) for readability. In this case, the integral in Eq 23 can be expressed as a two-dimensional convolution in space:

$$\begin{array}{c} \eta^{-1}\frac{d}{dt}\bar{w}\left(r\right)=\rho \int dr^{\prime }\;C\left(r-r^{\prime }\right)\bar{w}\left(r^{\prime }\right)-a\bar{w}\left(r\right)+b\;. \end{array}$$ (24)

**Fig 3:**
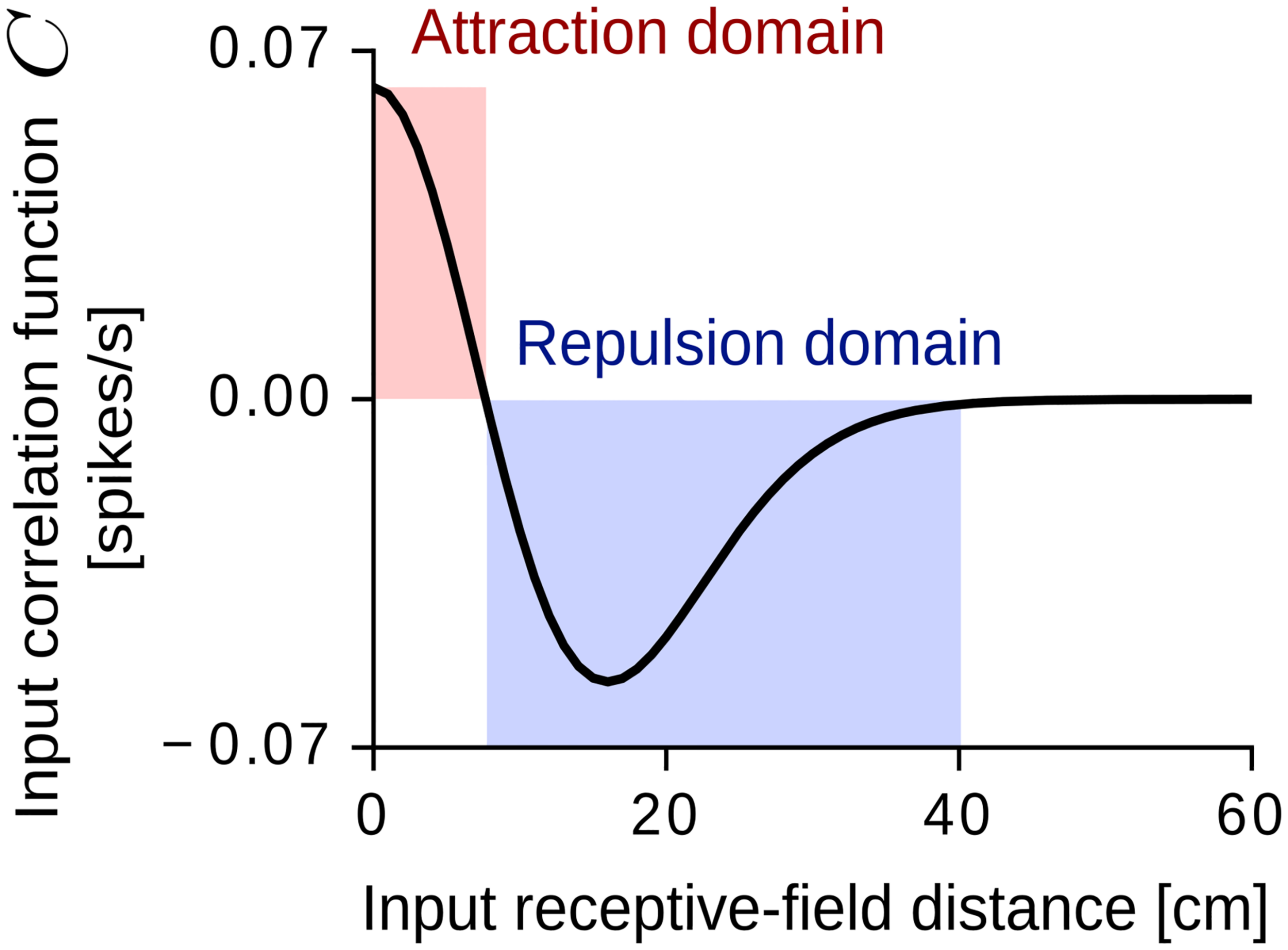
Input correlation function *C* for spatially-regular inputs. The function is circularly symmetric, i.e., it depends only on the distance |**r** − **r**′| between the receptive-field centers **r** and **r**′ (Eq 54). In the attraction domain (red shaded area) the correlation is positive and the synaptic weights grow in the same direction. In the repulsion domain (blue shaded area) the correlation is negative and the synaptic weights grow in opposite directions. Parameter values: *σ* = 6.25 cm, *r*_av_ = 0.4 s^−1^, *τ*_S_ = 0.1 s, *τ*_L_ = 0.16 s, *μ* = 1.06, *W*_tot_ = 1 s, *L* = 1 m, *v* = 0.25 m/s.

Fig 3 shows the correlation *C* as a function of the input receptive-field distance |**r** − **r**′| for the adaptation kernel *K* in Fig 1 and Gaussian input fields with size *σ* = 6.25 cm (Eq 9). The function *C* has the shape of a typical Mexican-hat kernel, i.e., it is positive for short receptive-field distances (attraction domain), negative for intermediate distances (repulsion domain), and zero otherwise. In this case, the synaptic weights of close-by input fields grow together whereas the synaptic weights of input fields that are further apart are repelled from each other (Eq 24). Such a competitive Mexican-hat interaction is at the basis of many pattern-forming systems found in nature, and it is directly related to diffusion-driven instabilities of the Turing type [see e.g. 87].

#### Eigenvalue spectrum for spatially-regular inputs

To study spatially-periodic solutions, we take the two-dimensional Fourier transform with respect to **r** at both sides of Eq 24:

$$\begin{array}{c} \eta^{-1}\frac{d}{dt}\hat{w}\left(k\right)=\left(\rho \hat{C}\left(k\right)-a\right)\;\hat{w}\left(k\right)+\delta \left(k\right)b \end{array}$$ (25)

where we defined the Fourier transform pair

$$\begin{array}{c} \hat{w}\left(k\right)≔\int \;dr\;\bar{w}\left(r\right)\exp\left(-2\pi j\;k\cdot r\right)\;\;,\;\;\bar{w}\left(r\right)=\frac{1}{{\left(2\pi \right)}^{2}}\int \;dk\;\hat{w}\left(k\right)\exp\left(2\pi j\;k\cdot r\right)\;, \end{array}$$ (26)

**k** is a two-dimensional wave vector, and $j=\sqrt{-1}$ is the imaginary unit. Solving Eq 25 for **k** ≠ (0, 0), we obtain

$$\begin{array}{c} \hat{w}\left(k\right)={\hat{w}}_{0}\left(k\right)\exp\left(\eta \lambda \left(k\right)t\right) \end{array}$$ (27)

where ${\hat{w}}_{0}\left(k\right)$ denotes the weight spectrum at time *t* = 0, and we defined

$$\begin{array}{c} \lambda \left(k\right)≔\rho \hat{C}\left(k\right)-a\;\text{for}\;k\neq \left(0,0\right)\;. \end{array}$$ (28)

The function λ(**k**) defines the eigenvalue spectrum of the dynamical system in Eq 24, and the corresponding eigenfunctions are the elements of the Fourier basis exp(2*πj* **k** ⋅ **r**). Eq 28 is also called the *dispersion relation* of the system. Note that solving Eq 25 for **k** = (0, 0) one obtains the dynamics of the total synaptic weight, which is kept normalized by the learning rule (Sec Weight normalization).

From Eq 27, the Fourier modes of the synaptic weights $\hat{w}\left(k\right)$ grow or decay exponentially with rates proportional to the eigenvalues λ(**k**). Therefore, a structure in the synaptic weights emerges on a time scale

$$\begin{array}{c} \tau_{\text{str}}≔\frac{1}{\eta \lambda_{\text{max}}} \end{array}$$ (29)

where λ_max_ ≔ max_**k**_[λ(**k**)] is the largest eigenvalue in the system.

Importantly, the eigenvalues λ(**k**) are linearly related to the Fourier transform of the input-correlation function $\hat{C}\left(k\right)$ (Eq 28), which is circularly-symmetric for circularly-symmetric inputs. In this case, in Sec Input correlation for spatially-regular inputs (Eq 62) we derive

$$\begin{array}{c} \hat{C}\left(k\right)\approx \frac{W_{\text{tot}}}{L^{2}}\;4\pi^{2}{\tilde{G}}^{2}\left(k\right)\;{\tilde{K}}_{\text{sp}}\left(k\right)\;\;\text{with}\;k≔\left|k\right| \end{array}$$ (30)

where $\tilde{G}$ and ${\tilde{K}}_{\text{sp}}$ (Eqs 63 and 64) are the zeroth-order Hankel transforms (Eq 59) of the input tuning curve G (Eq 9) and of the equivalent adaptation kernel in space

$$\begin{array}{c} K_{\text{sp}}\left(r\right)\;\overset{\left(61\right)}{≔}\;\frac{1}{rv}K\;(\frac{r}{v})\;. \end{array}$$ (31)

Finally, by plugging Eqs 30 into 28, we obtain

$$\begin{array}{c} \lambda \left(k\right)\approx \rho \frac{W_{\text{tot}}}{L^{2}}\;4\pi^{2}{\tilde{G}}^{2}\left(k\right)\;{\tilde{K}}_{\text{sp}}\left(k\right)-a\;\text{with}\;k\neq 0\;. \end{array}$$ (32)

From Eqs 27 and 32 we recognize a necessary condition for spatial patterns to emerge: the eigenvalue spectrum λ(**k**) = λ(*k*) shall have a global maximum λ_max_ > 0 at a frequency *k*_max_ > 0. In this case, all the Fourier modes **k** at the critical frequency |**k**| = *k*_max_ are unstable (Eq 25), and spatially-periodic patterns could emerge.

**Fig 4:**
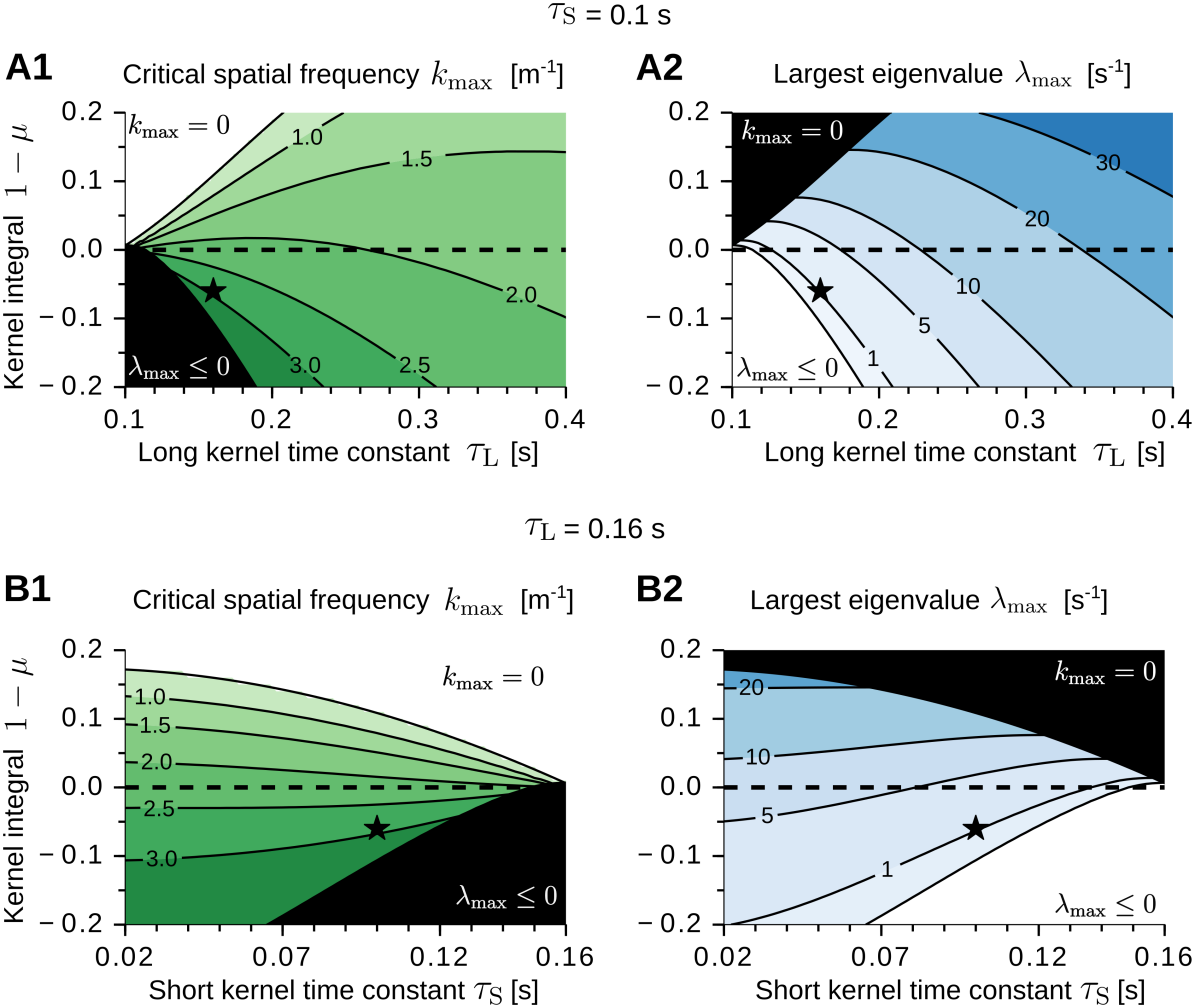
Impact of the adaptation kernel on grid-pattern formation. (A1-A2) Critical spatial frequency *k*_max_ (A1) and largest eigenvalue λ_max_ (A2) as a function of the kernel integral 1 − *μ* and the long kernel time constant *τ*_L_. The short time constant is *τ*_S_ = 0.1 s. The black lines are iso-levels (see annotated values). Regions enclosed by two adjacent iso-lines are colored uniformly (darker colors denote larger values). Within the black region in A1 we obtain λ_max_ ≤ 0 s^−1^ (see white region in A2). Within the black region in A2 we obtain *k*_max_ = 0 m^−1^ (see white region in A1). The dashed horizontal line indicates zero-integral kernels. The star denotes the parameter values *τ*_S_ = 0.1 s, *τ*_L_ = 0.16 s, *μ* = 1.06 of the kernel in Fig 1. (B1-B2) Same as in A but varying the short kernel time constant *τ*_S_. The long time constant is *τ*_L_ = 0.16 s. The eigenvalue spectrum is estimated from Eq 32. Further parameter values: *σ* = 6.25 cm, *r*_av_ = 0.4 s^−1^, *W*_tot_ = 1 s, *ρ* = 900 m^−2^, *L* = 1 m, *v* = 0.25 m/s, *a* = 1.1 s^−1^.

Fig 4 shows the critical frequency *k*_max_ (panels A1 and B1) and the largest eigenvalue λ_max_ (panels A2 and B2) as a function of the parameters of the adaptation kernel *K*, i.e., the short time constant *τ*_S_, the long time constant *τ*_L_, and the kernel integral 1 − *μ* (Eq 2). The input receptive-field width *σ* is kept constant. In panels A1 and B1, the green-shaded regions correspond to parameter values where periodic grid-like patterns could emerge (*k*_max_ > 0). Conversely, the white regions denote parameter values where place-cell-like receptive fields could emerge (*k*_max_ = 0) [88]. We note that the spatial scale of the periodic patterns depends on the long adaptation time constant *τ*_L_ (panel A1), but is largely unaffected by the short time constant *τ*_S_ (panel B1). Additionally, the largest spatial frequencies are obtained for small values of *τ*_L_ and negative kernel integrals (panel A1). This leads us to the following predictions: the grid scale shall depend on the long temporal dynamics of the adaptation kernel, and the smallest grid scales require adaptation kernels with an overall inhibitory effect on the activity of the output neuron. We also note that larger values of *τ*_L_ correspond to larger values of λ_max_ (panel A2). Thus, we predict that grids at larger scales shall develop faster than grids at smaller scales (Eq 29).

Importantly, the formation of grid-like patterns also requires a nonlinearity in the system. Indeed, for triangular lattices to emerge, only three wave vectors **k** of the same length |**k**| shall survive. But this cannot be achieved in a linear system where all Fourier modes develop independently from each other (Eq 27). Yet the non-linear weight constraints imposed in our model (Eq 6) are sufficient to generate triangular patterns (Sec Emergence of grid spatial patterns).

In summary, the theory presented here gives necessary conditions for spatial pattern formation, and it predicts how the shape of the adaptation kernel *K* influences the scale of the grids and the relative time required for their formation. The theory remains however agnostic about the specific two-dimensional periodicity of the resulting patterns, i.e., it cannot predict whether the final solutions are, e.g., planar waves, square, rhomboidal, or triangular lattices. Further mathematical insights on this topic could be obtained by using perturbation methods [see e.g. 89], but this is beyond the scope of the current manuscript.

### Numerical results on grid-pattern formation

In Sec Mathematical results on grid-pattern formation we derived an equation for the average dynamics of the synaptic weights *w*_*i*_, under the STDP learning rule and the stochastic activation of input and output neurons (Eq 16). In the case of spatially-regular inputs, we then computed the systems eigenvalue spectrum λ(*k*) in terms of the Gaussian input tuning curve G and the temporal adaptation kernel *K* (Eq 32). We showed that periodic spatial patterns could emerge if the eigenvalue spectrum λ(*k*) had a global maximum λ_max_ > 0 at a frequency *k*_max_ > 0 (Fig 4).

**Fig 5:**
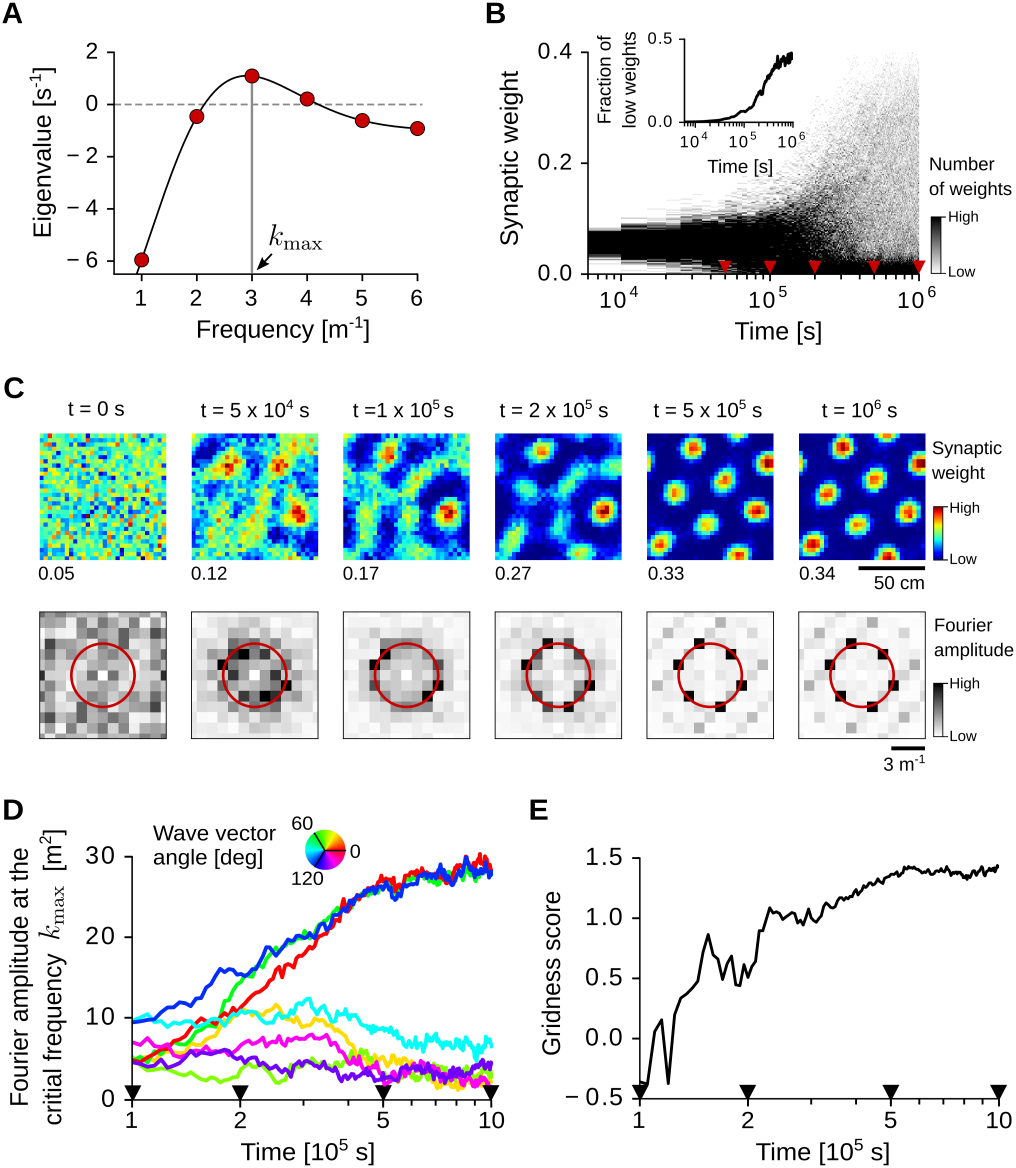
Grid-pattern formation with spatially-regular inputs. (A) Eigenvalue spectrum λ(*k*) of the averaged weight dynamics (Eq 32). The black solid line shows the continuous spectrum in the limit of infinite-size environments; the red dots show the discrete eigenvalues for a square arena of side length *L* = 1 m with periodic boundaries. The horizontal dashed line separates positive and negative eigenvalues. The vertical gray line indicates the critical spatial frequency *k*_max_ = 3 m^−1^. The eigenvalue at frequency *k* = 0 is not shown. Parameter values: *τ*_S_ = 0.1 s, *τ*_L_ = 0.16 s, *σ* = 6.25 cm. (B) Time-resolved distribution of *N* = 900 synaptic weights updated according to the STDP rule in Eqs 3–6. Red triangles indicate the time points shown in C. Inset: fraction of weights close to the lower saturation bound (*w_i_* < 5 ⋅ 10^−3^). (C) Top row: evolution of the synaptic weights over time. Weights are sorted according to the two-dimensional position of the corresponding input receptive-field centers. Note that each panel has a different color scale (maximum weight at the bottom-left corner, see B for distributions). Bottom row: Fourier amplitude of the synaptic weights at the top row. The red circle indicates the frequency *k*_max_ = 3 m^−1^ of the largest eigenvalue (see panel A). (D) Time evolution of weights' Fourier amplitudes $\left|\hat{w}(k)\right|$ for wave vectors **k** at the critical frequency |**k**| = *k*_max_. Wave vector angles (color coded) are relative to the largest mode at the end of the simulation (*t* = 10^6^ s). The black triangles indicate time points in C. (E) Gridness score of the weight pattern over time. The gridness score quantifies the degree of triangular periodicity. See Sec Numerical simulations for further details and parameter values.

Fig 5A shows the eigenvalue spectrum λ(*k*) for a choice of the parameter values such that this condition is satisfied. With adaptation time constants *τ*_S_ = 0.1 s and *τ*_L_ = 0.16 s (Eq 2, Fig 1, star in Fig 4), and Gaussian input receptive fields of size *σ* = 6.25 cm (Eq 9), the eigenvalue spectrum peaks at the critical frequency *k*_max_ = 3 m^−1^. In the following, we simulate the emergence of grid-like patterns in this scenario.

#### Emergence of grid spatial patterns

First, we simulate the detailed spiking model with spatially-regular inputs (Sec Spatially-regular inputs). The results are shown in Fig 5B–5E. In line with the theory, a structure emerges in the synaptic weights (Fig 5B and 5C) on a time scale of *τ*_str_ = 1/(*η*λ_max_) ≈ 5 ⋅ 10^4^ s (Eq 29) where *η* = 2 ⋅ 10^−5^ is the learning rate and λ_max_ ≈ 1 s^−1^ is the largest eigenvalue in the system. Additionally, the weight spectrum is quickly dominated by the critical frequency *k*_max_ = 3 m^−1^ (Fig 5C, bottom row) at which the eigenvalue spectrum has a global maximum (Fig 5A).

Importantly, the synaptic weights also develop a periodic triangular symmetry, which is reminiscent of grid-cell patterns. Such triangular symmetry emerges after a substantial fraction of weights has hit the low saturation bound (Eq 6, Fig 5B, inset). Periodic pattern formation is indeed a strictly non-linear phenomenon, and excluding the spike generation process, weight saturation is the only non-linearity present in the system. In the linear regime, all Fourier modes **k** with frequency |**k**| = *k*_max_ exponentially grow with equal rate *η*λ_max_ and *independently* from each other (Eq 25). In this case, the random weight pattern at time *t* = 0 s is amplified at the frequency *k*_max_, but no periodic structure emerges. In the non-linear regime, instead, the exponentially growing modes are mutually coupled, and a spontaneous symmetry breaking occurs: only three Fourier modes with wave vectors that are 60 degrees apart survive in our simulations (see Fig 5C and 5D).

**Fig 6:**
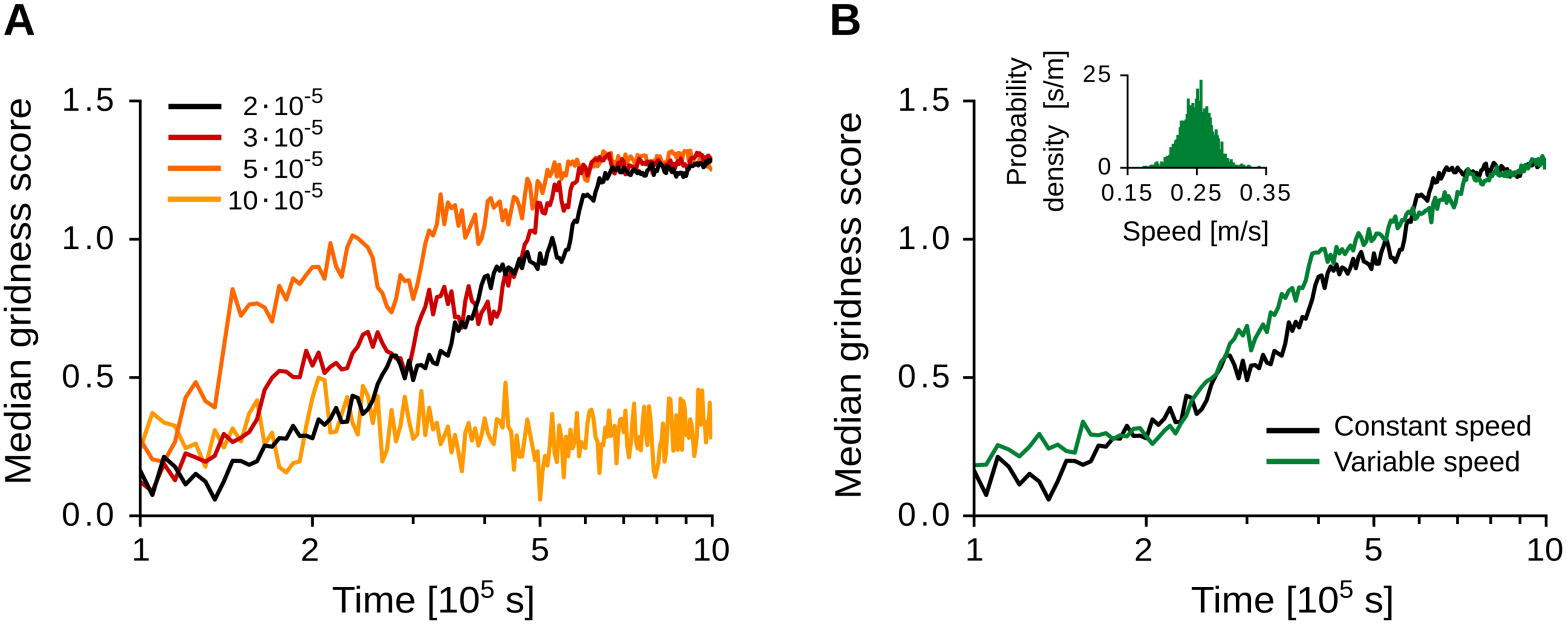
Time scales of learning. (A) Median gridness scores of the input synaptic weights for 40 random weight initializations and different learning-rate values, i.e., *η* = (2, 3, 5, 10) ⋅ 10^−5^. The weight development is simulated with the detailed spiking model with spatially-regular inputs and constant virtual-rat speed (see also Fig 5). (B) Median gridness scores of the input synaptic weights simulated with constant (black line) and variable (green line) virtual-rat speeds for 40 random weight initializations. Variable running speeds are obtained by sampling from an Ornstein-Uhlenbeck process with long-term mean $\bar{v}=0.25$ m/s, volatility *σ*_*v*_ = 0.1 m ⋅ s^−1.5^ and mean-reversion speed *θ*_*v*_ = 10 s^−1^. The inset shows the distribution of running speeds (mean: 0.25 m/s std: 0.02 m/s). Note that the long-term mean $\bar{v}$ of the process equals the speed *v* in constant-speed simulations. See Sec Numerical simulations for further details and additional parameter values.

In the example of Fig 5, a triangular symmetry starts to emerge after 2 ⋅ 10^5^ s, i.e., about 50 hours of exploration of the virtual rat. Yet the time scale of learning depends on the learning rate *η* and on the largest eigenvalue λ_max_ (Eq 29), which are under-constrained by experimental data (see Eq 32 for the dependence of λ_max_ on other model parameters). From a theoretical standpoint, the speed of learning is limited by the noise in the system, which is due to the virtual-rat random walk and the stochastic spiking of the neurons. To theoretically explore this limit and test the robustness of the model against noisy initial conditions, we simulated the development of the synaptic weights for different values of the learning rate and multiple random initializations of the synaptic weights. The results are reported in Fig 6A. With larger learning rates, grid-like patterns emerge faster. However, if the learning rate is too large, e.g., *η* = 10 ⋅ 10^−5^ in our simulations, the gridness score fluctuates at low levels and no stable grid pattern emerges (yellow line in Fig 6A). Therefore, our results suggest that tens of hours of spatial exploration are required for stable grid patterns to emerge. Finally, the model is robust to random initializations of the synaptic weights, and to variations of the running speed of the virtual rat (Fig 6B).

#### Geometrical properties of the grid patterns

We now discuss the geometrical properties of the simulated grid patterns. A periodic triangular grid is characterized by three fundamental properties: i) the grid *scale*, i.e., the distance between two neighboring peaks; ii) the grid spatial *phase*, i.e., the spatial offset of the grid peaks with respect to a reference point; and iii) the grid *orientation*, i.e, the angle between one of the three grid axes and a reference direction.

#### Grid scale

**Fig 7:**
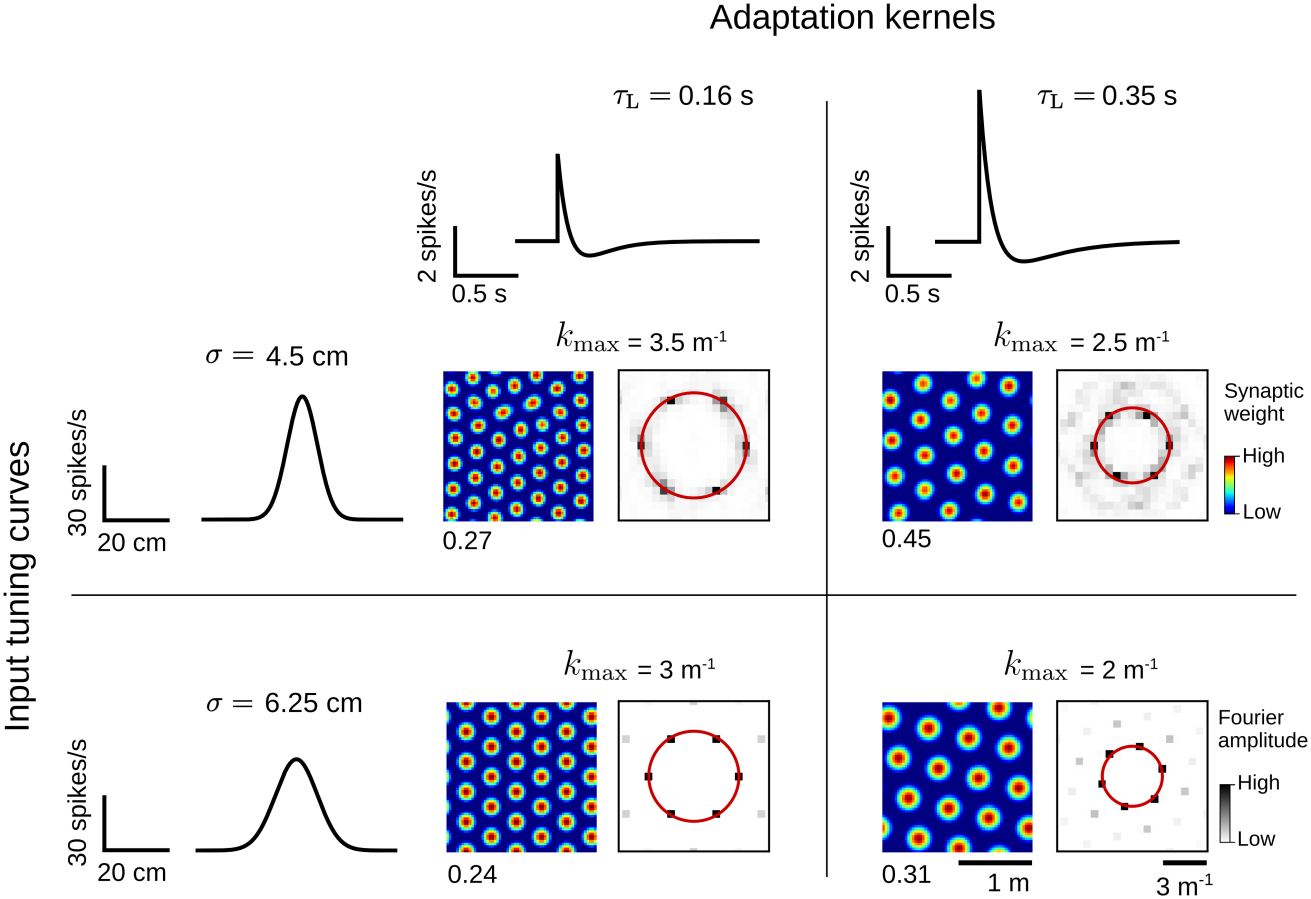
Spatial scale of the grid patterns. Example grid patterns obtained with different adaptation kernels *K* (Eq 2, top row) and different input tuning curves G (Eq 9, left-most column). For each choice of the functions *K* and G, the synaptic weights (left) and their corresponding Fourier spectra (right) at the end of the simulation are shown (*t* = 10^6^ s). The synaptic-weight maps have different color scales (maximal values at the bottom-left corner). The red circles indicate the spatial frequency *k*_max_ of the weight patterns. Synaptic weights were obtained by simulating the average weight dynamics in Eq 16. Note that we used a larger enclosure (*L* = 2 m) as compared to the one in Figs 5 and 6 (*L* = 1 m). See Sec Numerical simulations for further details and parameter values.

In our model, the grid scale is set by the critical frequency *k*_max_ at which the eigenvalue spectrum has a global maximum (Eq 32 and Fig 5). This critical frequency depends only on the movement speed *v* of the virtual rat, the width *σ* of the input tuning curve G, and the temporal dynamics of the adaptation kernel *K* (Fig 4). Therefore, grid patterns at different scales are obtained, for example, by varying the width *σ* of the input receptive fields or the long time scale *τ*_L_ of the adaptation kernel (Fig 7, see also Fig 4). This theoretical result is consistent with the facts that spatial tuning in the hippocampal formation is typically broader ventrally than dorsally [90–92], and that grid scales vary in the same direction [1, 93]. Additionally, we predict that the adaptation time scale may also have a dorso-ventral gradient, similarly to other intrinsic cellular properties in the mEC [e.g. 71, 94–97].

#### Grid spatial phase

**Fig 8:**
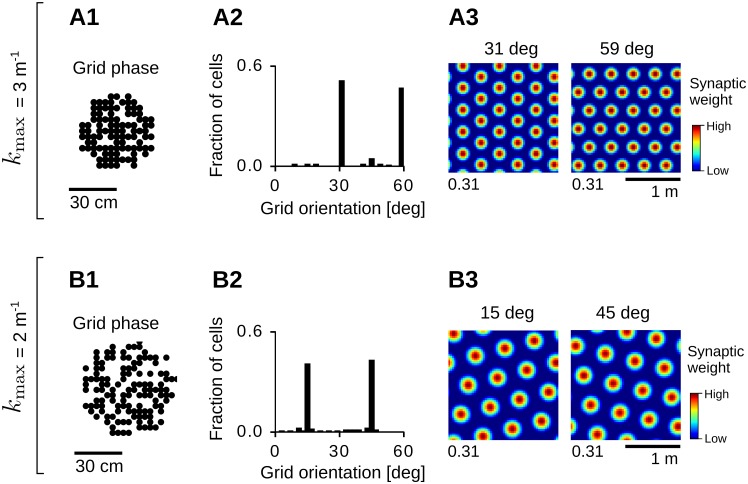
Geometric properties of the grid patterns. (A) Distribution of grid spatial phases (A1) and grid orientations (A2) for patterns at frequency *k*_max_ = 3 m^−1^ in an arena of side-length *L* = 2 m (*σ* = 6.25 cm, *τ*_L_ = 0.16 s; see also Fig 7, bottom-left panel). Distributions were obtained from the average weight dynamics in Eq 16 for 200 random initializations of the synaptic weights (*t* = 10^6^ s). Only patterns with gridness scores larger than 0.5 were considered (197/200). Panel A3 shows example weight patterns for the two most common orientations in A2 (maximal values at the bottom-left corner). (B) Same as in A but for patterns at spatial frequency *k*_max_ = 2 m^−1^ in an arena of side-length *L* = 2 m (*σ* = 6.25 cm, *τ*_L_ = 0.35 s; see also Fig 7, bottom-right panel). A fraction of 182/200 grids had a gridness score larger than 0.5. See Sec Numerical simulations for further details and parameter values.

With evenly-distributed input fields and periodic boundaries, the spatial phases of the grid patterns depend only on the initial condition of the synaptic weights, i.e., random weight initializations result in uniformly-distributed grid phases (Fig 8A1 and 8B1). This result is in line with the phases of nearby grid cells being roughly evenly distributed in experimental data [1], but see also [98]. Yet it remains unclear whether the same results would be obtained in the case of non-periodic boundaries.

#### Grid orientation

**Fig 9:**
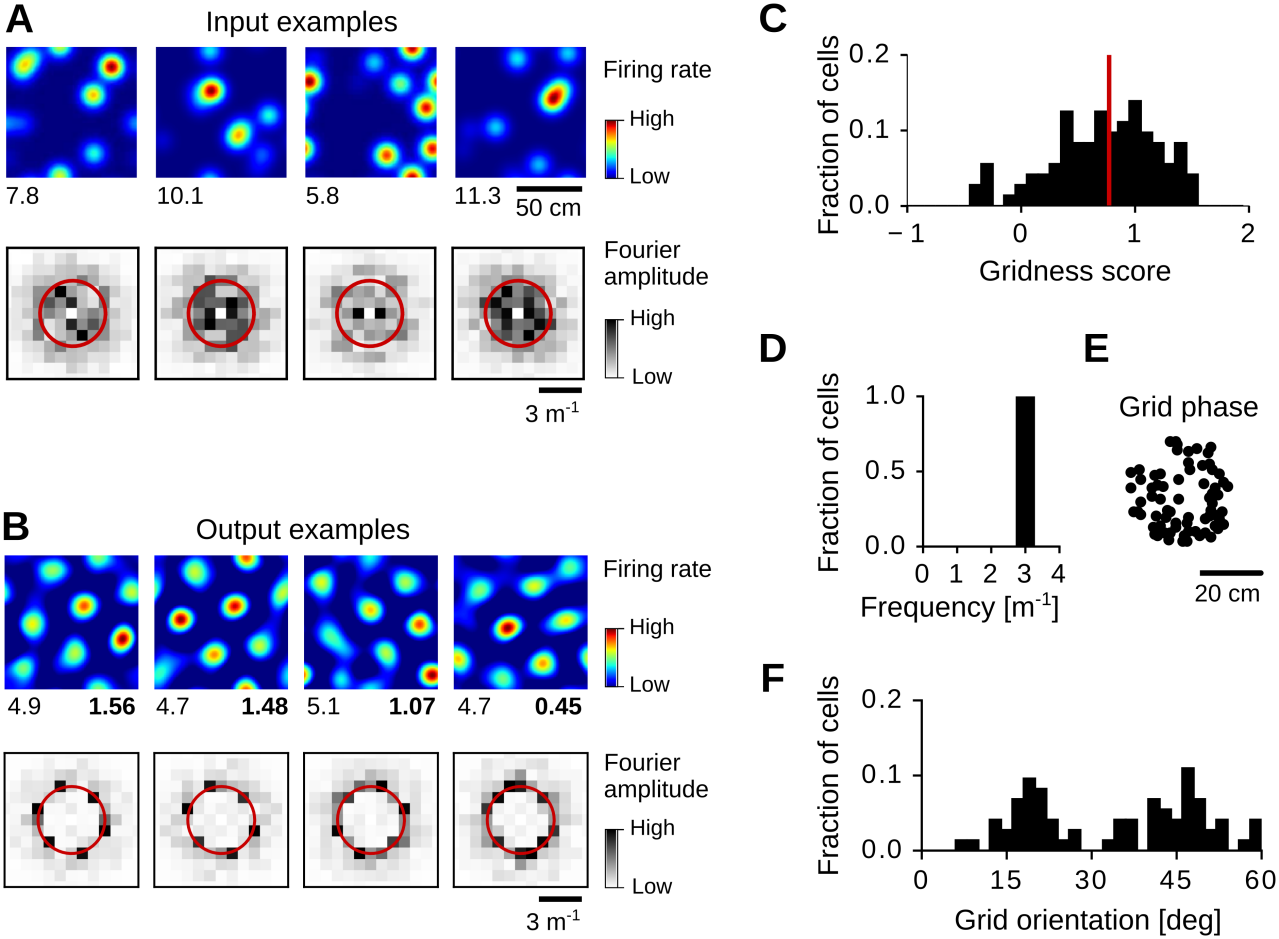
Grid-pattern formation with spatially-irregular inputs. (A) Four examples of irregular input firing-rate maps (top row) and the corresponding Fourier spectra (bottom row). The maximal firing rate (spikes/s) is reported at the bottom-left corner. The red circles indicate the spatial frequency *k*_max_ = 3 m^−1^. (B) Four examples of output firing-rate maps (top row) and the corresponding Fourier spectra (bottom row). The gridness score is reported at the bottom-right corner. Output firing-rate maps were estimated from the average weight dynamics in Eq 16 (*t* = 10^6^ s) for four different realizations of the spatial inputs. (C-F) Distribution of gridness scores (C), grid spatial frequencies (D), grid spatial phases (E), and grid orientations (F) for 100 random realizations of the spatial inputs. The red vertical line in C indicates the mean score (0.77). See Sec Numerical simulations for further details and parameter values.

With periodic boundary conditions, our model produces grid orientations that are distributed non-uniformly. Precisely, the distribution of grid orientations depends on the scale of the pattern relative to the size of the environment, e.g., in the same environment patterns at different scales tend to align differently (compare panels A2 and B2 in Fig 8). In the examples of Fig 8A3, one of the grid axes tends to align to a border of the arena whereas in the examples of Fig 8B3 one of the grid axes tends to align to a diagonal of the arena. Similar results are obtained by keeping the grid scale fixed and varying the size of the environment, e.g., compare Fig 8A2 (*k*_max_ = 3 m^−1^ and *L* = 2 m) and Fig 9F (*k*_max_ = 3 m^−1^ and *L* = 1 m). In general, we expect grid orientations to be uniformly distributed only in infinite-sized environments, or in environments that are much larger than the pattern size. Nevertheless, because grid orientation depends on the boundary conditions, it remains difficult to compare the distributions obtained here with the ones observed experimentally [1, 93, 99, 100]. Finally, in order to explain grid alignment across cells and/or environments [1, 93], collateral interactions between developing grid cells may be required [50, 51, 101] (see also Sec Recurrent dynamics).

#### Pattern formation with spatially-irregular inputs

We demonstrated the emergence of grid-like patterns in the case of *spatially-regular* inputs, i.e., for each input cell having a single Gaussian receptive field in space (Sec Spatially-regular inputs). We now show that similar results are obtained in the case of *spatially-irregular* inputs (Sec Spatially-irregular inputs). We generate spatially-irregular inputs $\Psi_{i}^{\text{in}}$ by superimposing *M* > 1 Gaussian receptive fields with equal width *σ*, but random centers and random amplitudes (Eq 10, see Fig 9A for examples). The functions $\Psi_{i}^{\text{in}}$ are normalized such that their average firing rate *r*_av_ is constant for all input neurons and independent from the number *M* of superimposed receptive fields.

We test grid-pattern formation in this scenario by simulating the average dynamics of the synaptic weights (Eqs 16 and 21) for random realizations of the input tuning curves $\Psi_{i}^{\text{in}}$, with *N* = 3600 input neurons and *M* = 10 receptive fields per neuron. We then estimate output firing-rate maps from the synaptic weights at the end of the simulations (*t* = 10^6^ s). The results are shown in Fig 9B and 9C. In the majority of the cases (73/100) a regular grid-like pattern emerges at the output.

Like in the case of spatially-regular inputs, the spatial scale of the output patterns depends on the long adaptation time constant *τ*_L_ and on the width *σ* of the input receptive fields. Indeed, for *σ* = 6.25 cm and *τ*_L_ = 0.16 s, we obtain output grid patterns with spatial frequency *k*_max_ = 3 m^−1^ (Fig 9D), which is equal to the one obtained for spatially-regular inputs with the same parameter values (Figs 5 and 7, bottom-left panel). This can be understood by the fact that the expected eigenvalue spectrum for spatially-irregular inputs 〈λ_irr_(*k*)〉 is qualitatively similar to the eigenvalue spectrum λ(*k*) for spatially-regular inputs (Sec Eigenvalue spectrum for spatially-irregular inputs, Eq 72):

$$\begin{array}{c} \langle \lambda_{\text{irr}}\left(k\right)\rangle \approx \Phi \lambda \left(k\right)+\text{const}. \end{array}$$ (33)

where 0 ≤ Φ ≤ 1 is a scale factor. We also find that the scale factor Φ depends on the number *M* of superimposed fields, i.e., Φ ≈ 4/(3*M*) for *M* > 3 (Eq 82), meaning that structure formation is slower for larger numbers of superimposed fields (Eq 29).

Finally, like in the case of spatially-regular inputs, with periodic boundary conditions the spatial phases of the simulated grids distribute evenly in the arena (Fig 9E), and the grid orientations tend to cluster according to the grid scale and the size of the environment (Fig 9F, see also Fig 8A2 for the same grid scale in a larger environment).

## Discussion

We studied the origin of grid-cell patterns in a single-cell spiking model relying solely on 1) spatially-tuned feed-forward inputs, 2) spike-rate adaptation, 3) and synaptic plasticity at the input synapses. We considered two input scenarios: spatially-regular inputs (reminiscent of place-cell activity), and spatially-irregular inputs (reminiscent of parasubicular activity). First, we studied the average dynamics of the system analytically, and we derived necessary conditions for the emergence of spatially-periodic solutions (Sec Mathematical results on grid-pattern formation). We then simulated the model numerically, and showed that grid-like patterns emerge both with spatially-regular and spatially irregular inputs (Sec Numerical results on grid-pattern formation). In the following, we discuss the main assumptions and predictions of our model.

### Input spatial tuning and the origin of grid-cell patterns

We assumed that the feed-forward input activity is spatially tuned. Such spatial tuning could be provided by hippocampal place cells, or by other cortical or sub-cortical structures with less regular spatial firing. From a theoretical point of view, we find that grid patterns emerge faster with place-cell-like inputs, i.e., with inputs having a single receptive field in space. From an anatomical point of view, both scenarios seem plausible. On the one hand, grid-cell activity requires excitatory drive from the hippocampus [102], which projects to the deep layers of the mEC [103, 104] where grid cells are found [23, 60]. On the other hand, parasubicular inputs target layer II of the mEC [61, 105–108] where grid cells are most abundant [23, 60]. Although a small fraction of parasubicular cells already shows grid-like tuning [60, 61], the activity in parasubiculum is often characterized by multiple spatially-irregular fields [57–61] similar to those assumed in our model (Fig 9A).

That grid-cell activity could originate from parasubicular inputs is further supported by the detailed layout of the entorhinal circuit. Layer II principal neurons segregate into stellate and pyramidal cells, which are distinguished by their morphology, intrinsic properties [69], and immunoreactivity [109–111]. Interestingly, pyramidal-cell somata cluster into anatomical patches [110, 111], which are preferentially targeted by parasubicular axons [61]; and the spiking activity in parasubiculum precedes the activity of layer II pyramidal cells by a few degrees in the theta cycle [61]. Such a network configuration suggests that grid patterns may originate in the layer II pyramidal cells via parasubicular inputs, and be inherited by the stellate cells via feed-forward projections. Consistent with this view is that both stellate and pyramidal cells show grid spatial tuning [55], and that direct intra-laminar connections are found from pyramidal onto stellate cells and not vice-versa [112, 113]; but see [114].

In summary, our model is consistent with entorhinal grid-cell activity originating either in the superficial layers via parasubicular input or in the deep layers via hippocampal input. It is also possible that multiple sites of origin exist, and that grid-like tuning is inherited—and even sharpened—via feed-forward projections from the deep to the superfical layers [115–119] or from the superficial to the deep layers [104].

### Spike-rate adaptation

Our grid-cell model relies on the presence of a spike-rate adaptation mechanism. Spike-rate adaptation has been observed throughout the cortex [62], and is prominent in layer II of the mEC, in both stellate and pyramidal neurons [69, 70]. Yoshida et al. [71] also reported a dorso-ventral gradient in the adaptation strength of layer II entorhinal cells. However, because adaptation was found to be stronger ventrally than dorsally, Yoshida et al. [71] interpreted their results as evidence against grid-cell models based on adaptation. Yet the critical variable controlling the grid scale is not the strength of adaptation, but rather its temporal dynamics (Fig 7), which was not systematically analyzed [71]; see also [101] for a similar discussion on this point.

We modeled spike-rate adaptation by applying a temporal kernel *K* to the input spike trains (Eq 1). The kernel *K*, was composed of a brief depolarization peak and a slower hyper-polarizing potential (on a time scale of hundreds of milliseconds). Such a slow hyper-polarizing potential reduced the output firing rate in response to persistent excitation, and it filtered the input activity in a low-frequency band (i.e. with a resonance frequency of about 1 Hz, see Fig 1). The shape of the kernel was motivated by long-lasting hyper-polarizing potentials following excitatory post-synaptic potentials found in hippocampal CA1 pyramidal neurons [120], although similar responses have not been observed in the mEC yet.

However, the formation of grid-cell patterns could rely on any other cellular or synaptic mechanism that effectively acts as a band-pass filter on the input activity. A candidate mechanism is the after-spike hyperpolarizing potential (AHP). AHPs are indeed observed in the superficial layers of the mEC where single action potentials are followed by both a fast (2-5 ms) and a medium AHP (20-100 ms) [69, 97, 121]. To assess whether such hyperpolarizing potentials could underlie grid-pattern formation, we extended our model to account for AHPs (Sec Pattern formation with after-spike potentials). However, we found that grids at typical spatial scales cannot be obtained by AHPs alone. Yet after-spike potentials could amplify the effects of a band-pass filtering mechanism that is already present at the input.

Spike-rate adaptation could also rely on hyperpolarization-activated cation currents (*I*_h_), which depend on HCN channels [122, 123]. Fast *I*_h_ currents (mediated by HCN1 channels) have been shown to control the theta-frequency resonance of entorhinal stellate cells in vitro [95, 97, 124–126]. Instead, slower *I*_h_ currents (mediated by HCN2-4 channels) could generate in entorhinal cells the low-frequency resonance assumed by our model (Fig 1B).

### Synaptic plasticity

We propose that grid-cell patterns emerge from a synaptic reorganization of the mEC network, which is assumed to be plastic. This is in line with both LTP and LTD being reported in the entorhinal cortex [121, 127–130], but see also [131]. Additionally, asymmetric STDP was observed in the mEC [76]. Although we used a symmetric learning window in our model, the exact window shape has little effect on grid-pattern formation, provided that its temporal width (on the order of tens of milliseconds) is much shorter than the correlation length of the input activities (on the order of hundreds of milliseconds).

Structure formation via Hebbian learning is typically a slow process. In our model, grid-like patterns emerge on a time scale that is inversely proportional to the learning rate *η* and to the maximal eigenvalue λ_max_ (Eq 29). The latter depends on the spatial density *ρ* = *N*/*L*^2^ of input receptive fields, on the integral *W*_tot_ of the learning window, on the shapes of the input-tuning curves G, and on the dynamics of the adaptation kernel *K* (Eq 32). Because most of these quantities are under-constrained by empirical data, a direct comparison with experimental time scales remains difficult. Yet learning shall be slow enough such that the input correlations that drive structure formation dominate over random fluctuations of the synaptic weights, which are due to the random walk of the virtual rat and the shot noise of the stochastic spiking. In our simulations, we find that this requires tens of hours of spatial exploration (Fig 6A).

Such slow process may seem in contrast with grid-cell activity appearing immediately in a novel environment [1, 132]. However, grid-like tuning may not need to be learned in each environment anew, but rather recalled—and possibly refined—from the experience of similar environments explored in the past. Although hippocampal place cells [133, 134] and entorhinal non-grid spatial cells [56] seem to remap completely in novel spaces, pattern formation could still leverage on residual correlations across environments that are hardly observable from the simultaneous recordings of only a few tens of neurons. Additionally, grid-cell learning could generalize across spatial contexts through border and boundary-vector inputs [84, 85], which are invariant across environments.

We suggest that a structure in the synaptic weights may be formed during the animal’s ontogenetic development, i.e., within a two-week period after the animal leaves the nest [135–137]. Consistent with this hypothesis is that stable spatial firing is observed before grid-cell maturation, e.g., hippocampal place cells develop prior to grid cells [135, 136] and irregular spatial cells are present before grid cells [137].

### Recurrent dynamics

We studied the emergence of grid patterns in a purely single-cell model, ignoring any network-level interaction between the neurons. However, because excitatory and inhibitory recurrent circuits have been described in the mEC [19, 20, 112, 113, 138], grid cells are likely to be mutually coupled [139, 140]. Such recurrent connections could explain the modular organization of grid-cell properties [93, 101] and their coherent responses to environmental changes [139]. Feedback interactions within a module may also amplify an initially broad grid-tuning given by the feed-forward inputs, similarly to the sharpening of receptive fields in visual cortex [141, 142]. Finally, recurrent dynamics may sustain grid-like activity when the feed-forward inputs are temporally untuned, like in attractor models [14]. Still, spatially-tuned feed-forward inputs could be required for the initial formation of grid-like patterns [see e.g. 21].

### Related models

Our work—and the one by Kropff and Treves [49]—belong to a broad category of grid-cell models based on spatially-tuned feed-forward inputs and Hebbian synaptic plasticity [63–67]. In all these models, periodic spatial patterns arise via a common underlying principle: the input correlations that drive the dynamics of the synaptic weights have the form of a Mexican-hat kernel (Fig 3). What distinguishes the models among each other—and generates distinct predictions—is the specific mechanism by which such Mexican-hat interactions are obtained.

In our model, a Mexican-hat kernel results from the intrinsic adaptation dynamics of the output neuron, which controls the grid scale directly (Fig 7).

By contrast, in the models by Castro and Aguiar [63] and Stepanyuk [64], Mexican-hat correlations arise from the learning rule itself, i.e., by assuming that synaptic plasticity switches between LTP and LTD based on pre- and post-synaptic activities [143]. In this case, the grid spatial scale shall be affected by interfering with the learning rule.

In a different model, Dordek et al. [65] obtain Mexican-hat correlations by constraining the input activity to be effectively zero-mean. The authors discuss that such a zero-mean constraint could originate either from lateral inhibition or from a zero-mean temporal filter controlling the output activity of the neuron. In the latter case, the model by Dordek et al. [65] is analogous to the present one. We note, however, that effectively zero-mean inputs are neither necessary nor sufficient for grid patterns to emerge. Instead, pattern formation depends on the dynamics of the temporal filter and on the shape of the input tuning curves, but not on their means. This can be easily understood by considering the system’s eigenvalue spectrum in Fourier space (Eq 30), where the zero-frequency mode (*k* = 0) is not relevant for the emergence of spatially-periodic patterns. Also note that the smallest grid scales in our model are obtained with negative-mean temporal filters (Fig 4). Yet our results agree with the ones of Dordek et al. [65] in that the non-linearity introduced by imposing non-negative synaptic weights is sufficient for a triangular symmetry to emerge.

Alternatively, Mexican-hat correlations could emerge from phase-precessing feed-forward inputs [66]. In this case, grid-cell activity shall be impaired when phase precession is disrupted.

Finally, Weber and Sprekeler [67] proposed a model where the interplay between spatially-narrow feed-forward excitation and spatially-broad feed-forward inhibition generates a Mexican-hat kernel. This model predicts that the grid scale shall be affected by manipulating inhibitory inputs to the mEC.

### Model predictions and conclusion

We presented a single-cell model for the origin of grid-cell activity based on Hebbian synaptic plasticity and spike-rate adaptation. Our work builds upon the model by Kropff and Treves [49] and improves its original formulation in several aspects: 1) grid-like patterns emerge form a purely single-cell mechanism independently of any network-level interaction; 2) neuronal activities are spike-based and stochastic; 3) the input synaptic weights are purely excitatory; 4) the dynamics of the synaptic weights is studied analytically and linked to classical Turing-like patterns.

The present model makes the following experimental predictions. First, grid-cell patterns shall be affected by disrupting synaptic plasticity during ontogenetic development, which is consistent with preliminary data from Dagslott et al. [144]. Second, adult grid-cell activity shall be influenced by systematic behavioral or environmental biases in the first weeks of spatial exploration, e.g., by rising animals in environments without boundaries or with non-zero surface curvature [52, 145]. Third, the grid scale shall be affected by three factors: 1) the spatial tuning-width of the feed-forward inputs; 2) the average speed of the rat during ontogenetic development; 3) the time constant of the recovery from spike-rate adaptation. Fourth, grids at larger scales shall develop faster as compared to grids at smaller scales (Fig 4).

We believe that manipulations of the intrinsic adaptation properties of single cells are key to distinguish our model from other feed-forward models based on Hebbian learning (Sec Related models). To this end, further experimental work shall be devoted to pinpoint the biophysical mechanisms underlying adaptation in the mEC. Extensions of the present model could also explain how the geometry of the enclosure affects grid-cell symmetry [99], and how grid-like tuning emerges in non-spatial contexts [146, 147].

To conclude, our study contributes to a better understanding of the fundamental principles governing grid-cell activity, and lays the groundwork for more biophysically-realistic grid-cell models.

## Materials and methods

### Weight normalization

Here we derive the dynamics of the mean synaptic weight $w_{\text{av}}=N^{-1}\sum_{i=1}^{N}{\bar{w}}_{i}\;$ for a neuron with *N* synapses and temporally-averaged weights ${\bar{w}}_{i}$. We recall the weight dynamics in Eq 16

$$\begin{array}{c} \eta^{-1}\frac{d}{dt}{\bar{w}}_{i}=\sum_{j=1}^{N}\;C_{ij}{\bar{w}}_{j}-a{\bar{w}}_{i}+b\;\text{with}\;{\bar{w}}_{i}\geq 0\;. \end{array}$$ (34)

By taking the average over the index *i* at both sides of Eq 34 we obtain

$$\begin{array}{c} \eta^{-1}\frac{d}{dt}w_{\text{av}}=\left(NC_{\text{av}}-a\right)w_{\text{av}}+b \end{array}$$ (35)

where we defined the mean correlation *C*_av_ ≔ *N*^−2^∑_*ij*_ *C*_*ij*_. Note that we used the property ∑_*j*_ *C*_*ij*_ = *NC*_av_ for all *i*, which holds true for translation-invariant inputs. Therefore, for *NC*_av_ < *a*, the mean weight *w*_av_ decays exponentially with time constant

$$\begin{array}{c} \tau_{\text{av}}≔\frac{1}{\eta (a-NC_{\text{av}})} \end{array}$$ (36)

to the normalization level

$$\begin{array}{c} w_{\text{av}}^{\infty }≔\frac{b}{a-NC_{\text{av}}}\;. \end{array}$$ (37)

### Input correlation for general inputs

In this section we estimate the input correlation matrix

$$\begin{array}{c} C_{ij}\overset{\left(20\right)}{\approx }W_{\text{tot}}\int_{0}^{\infty }\;d\tau \;\;K\left(\tau \right)\bar{r_{i}^{\text{in}}\left(t\right)r_{j}^{\text{in}}\left(t-\tau \right)}\;\text{with}\;i,j=1,\ldots ,N \end{array}$$ (38)

for general spatial tuning curves $\Psi_{i}^{\text{in}}$ and smooth movement trajectories of the virtual rat (Sec Model of spatial exploration). We start by computing the temporal average $\bar{r_{i}^{\text{in}}\left(t\right)r_{j}^{\text{in}}\left(t-\tau \right)}$ of the product between the input activities $r_{i}^{\text{in}}\left(t\right)$ and the delayed input activities $r_{j}^{\text{in}}\left(t-\tau \right)$. We assume that the stochastic process **X**_*t*_ controlling the virtual-rat trajectory (Eq 11) is ergodic with respect to the auto-covariance, i.e.,

$$\begin{array}{c} \frac{1}{T}\int_{0}^{T}\;dt\;\;x_{t}\;x_{t-\tau }=\langle X_{t},X_{t-\tau }\rangle \;\text{for}\;T\to \infty \end{array}$$ (39)

where the angular brackets denote statistical expectation. By using this ergodicity property (Eq 39) and the spatial tuning of the inputs (Eq 7), we derive

$$\begin{array}{c} \bar{r_{i}^{\text{in}}\left(t\right)r_{j}^{\text{in}}\left(t-\tau \right)}=\bar{\Psi_{i}^{\text{in}}\left(x_{t}\right)\Psi_{j}^{\text{in}}\left(x_{t-\tau }\right)}\approx \langle \Psi_{i}^{\text{in}}\left(X_{t}\right)\Psi_{j}^{\text{in}}\left(X_{t-\tau }\right)\rangle \;. \end{array}$$ (40)

Note that Eq 40 is only valid in an approximate sense because Eq 39 assumes *T* → ∞, but the averaging time window has finite length *T* ≪ *τ*_str_ where *τ*_str_ is structure-formation time constant (Eq 29). From Eq 40 follows

$$\begin{array}{ccc} \bar{r_{i}^{\text{in}}\left(t\right)r_{j}^{\text{in}}\left(t-\tau \right)} & \approx & \left\langle \Psi_{i}^{\text{in}}\left(X_{t}\right)\Psi_{j}^{\text{in}}\left(X_{t-\tau }\right)\right\rangle \end{array}$$ (41)

$$\begin{array}{ccc} & ≔ & \;\int \int \;dx\;dx^{\prime }\;\Psi_{i}^{\text{in}}\left(x\right)\Psi_{j}^{\text{in}}\left(x^{\prime }\right)\;p\left(x,t,x^{\prime },t-\tau \right) \end{array}$$ (42)

$$\begin{array}{ccc} & = & \;\int \int \;dx\;dx^{\prime }\;\Psi_{i}^{\text{in}}\left(x\right)\Psi_{j}^{\text{in}}\left(x^{\prime }\right)\;p\left(x^{\prime },t-\tau |x,t\right)p\left(x,t\right) \end{array}$$ (43)

$$\begin{array}{ccc} & = & \frac{1}{L^{2}}\;\int \int \;dx\;dx^{\prime }\;\Psi_{i}^{\text{in}}\left(x\right)\Psi_{j}^{\text{in}}\left(x^{\prime }\right)\;p\left(x^{\prime },t-\tau |x,t\right)\; \end{array}$$ (44)

where the integrals in Eqs 42–44 run over all positions in the environment (a square arena of side-length *L*), and *p*(**x**, *t*, **x**′, *t* − *τ*) is the joint probability density of the virtual rat being at position **x** at time *t* and at position **x**′ at time *t* − *τ*. From Eqs 43 to 44, we used the fact that, for large times *t*, the virtual rat has equal probability of being in any position **x**, i.e., *p*(**x**, *t*) = 1/*L*^2^.

Eq 44 shows that the temporal average $\bar{r_{i}^{\text{in}}\left(t\right)r_{j}^{\text{in}}\left(t-\tau \right)}$ can be estimated from the input tuning curves $\Psi_{i}^{\text{in}}$ and $\Psi_{j}^{\text{in}}$, and the conditional probability density *p*(**x**′, *t* − *τ*|**x**, *t*). This conditional probability density has not yet been solved for correlated random walks in two dimensions [148]. Nevertheless, an additional approximation is possible. Because the temporal average $\bar{r_{i}^{\text{in}}\left(t\right)r_{j}^{\text{in}}\left(t-\tau \right)}$ is weighted by the adaptation adaptation kernel *K*(*τ*) (Eq 38), and *K*(*τ*) is negligible for *τ* > *τ*_max_ ≈ 5*τ*_L_ (Eq 2), we are interested in the conditional probability *p*(**x**′, *t* − *τ*|**x**, *t*) only at lags *τ* < *τ*_max_. In this case, for movement trajectories that are sufficiently smooth, we can assume that in a time *τ* the virtual rat has moved to a position **x** at distance |**x** − **x**′| = *τv* from the initial position **x**′, that is

$$\begin{array}{c} p\left(x^{\prime },t-\tau |x,t\right)\approx \frac{\delta (|x-x^{\prime }|-\tau v)}{2\pi \tau v} \end{array}$$ (45)

where *v* is the speed of the virtual rat (Eq 11), and the denominator ensures that *∫* d**x**′*p*(**x**′, *t* − *τ*|**x**, *t*) = 1; see also Fig 2 for exemplary virtual-rat trajectories in this scenario. We now use Eq 45 in Eq 44, and let **z** ≔ **x**′ − **x**:

$$\begin{array}{ccc} \bar{r_{i}^{\text{in}}\left(t\right)r_{j}^{\text{in}}\left(t-\tau \right)} & \approx & \frac{1}{L^{2}}\underset{≕\;\oint_{|z|=\tau v}\;dz}{\underset{︸}{\int dz\;\frac{\delta (|z|-\tau v)}{2\pi \tau v}}}\;\underset{≕\;\Psi_{i}^{\text{in}}⋆\Psi_{j}^{\text{in}}{|}_{z}}{\underset{︸}{\int dx\;\;\Psi_{i}^{\text{in}}\left(x\right)\Psi_{j}^{\text{in}}\left(x+z\right)}}\;. \end{array}$$ (46)

From Eq 46, the temporal average $\bar{r_{i}^{\text{in}}\left(t\right)r_{j}^{\text{in}}\left(t-\tau \right)}$ is approximated by the integral of the spatial cross-correlation $\Psi_{i}^{\text{in}}⋆\Psi_{j}^{\text{in}}$ over a circle of radius *τv*. Finally, by using Eq 46 in Eq 20, we obtain

$$\begin{array}{c} C_{ij}\approx \frac{W_{\text{tot}}}{L^{2}}\int_{0}^{\infty }\;d\tau \;K\left(\tau \right)\;\oint_{|z|=\tau v}\;dz\;\;\Psi_{i}^{\text{in}}⋆\Psi_{j}^{\text{in}}{|}_{z}\;. \end{array}$$ (47)

### Input correlation for spatially-regular inputs

In this section we compute the input correlation function *C* and its Fourier spectrum $\hat{C}$ in the case of spatially-regular inputs (see Sec Weight dynamics for spatially-regular inputs). First, we rewrite the input correlation matrix *C*_*ij*_ in Eq 21 as a continuous function *C*(**r**, **r**′) by labeling neurons according to their receptive-field centers **r** and **r**′:

$$\begin{array}{c} C\left(r,r^{\prime }\right)\approx \frac{W_{\text{tot}}}{L^{2}}\int_{0}^{\infty }\;d\tau \;K\left(\tau \right)\;\oint_{|z|=\tau v}\;dz\;\;\Psi_{r}^{\text{in}}⋆\Psi_{r^{\prime }}^{\text{in}}{|}_{z}\; \end{array}$$ (48)

where $\Psi_{r}^{\text{in}}\left(x\right)≔G(|x-r|)$ is a Gaussian input tuning curve centered at position **r** (Eq 9). Because the inputs are translation invariant, the correlation function *C* depends only on the translation vector **u** ≔ **r** − **r**′:

$$\begin{array}{ccc} C\left(r,r^{\prime }\right)=C\left(u,0\right)=C\left(u\right) & \approx & \frac{W_{\text{tot}}}{L^{2}}\int_{0}^{\infty }\;d\tau \;K\left(\tau \right)\;\oint_{|z|=\tau v}\;dz\;\;\Psi_{u}^{\text{in}}⋆\Psi_{0}^{\text{in}}{|}_{z} \end{array}$$ (49)

$$\begin{array}{ccc} & = & \frac{W_{\text{tot}}}{L^{2}}\int_{0}^{\infty }\;d\tau \;K\left(\tau \right)\;\oint_{|z|=\tau v}\;dz\;\;\Psi_{0}^{\text{in}}⋆\Psi_{0}^{\text{in}}{|}_{u+z} \end{array}$$ (50)

where $\Psi_{0}^{\text{in}}\left(x\right)≔G\left(|x|\right)$ is the tuning curve centered at the origin **0** = (0, 0). Next, we substitute in Eq 50 the definition of the integral operator in Eq 46:

$$\begin{array}{ccc} C(u) & \approx & \frac{W_{\text{tot}}}{L^{2}}\int_{0}^{\infty }\;d\tau \;K\left(\tau \right)\;\int dz\;\frac{\delta (|z|-\tau v)}{2\pi \tau v}\;\;\Psi_{0}^{\text{in}}⋆\Psi_{0}^{\text{in}}{|}_{u+z}\;. \end{array}$$ (51)

It is easy to see that the auto-correlation of a Gaussian is still a Gaussian:

$$\begin{array}{c} \Psi_{0}^{\text{in}}⋆\Psi_{0}^{\text{in}}{|}_{u}=\frac{L^{4}r_{\text{av}}^{2}}{4\pi \sigma^{2}}\exp\;(-\frac{{\left|u\right|}^{2}}{4\sigma^{2}}) \end{array}$$ (52)

from which we derive

$$\begin{array}{c} \Psi_{0}^{\text{in}}⋆\Psi_{0}^{\text{in}}{|}_{u+z}=\frac{L^{4}r_{\text{av}}^{2}}{4\pi \sigma^{2}}[\exp\;(-\frac{{\left|u\right|}^{2}+{\left|z\right|}^{2}}{4\sigma^{2}})\;\exp\;(-\frac{\left|u||z|\cos(\varphi \right)}{2\sigma^{2}})] \end{array}$$ (53)

where *φ* is the angle between the vectors **u** and **z**. Finally, by expressing in polar coordinates the vector **z** ≔ |**z**|[cos(*φ*), sin(*φ*)], from Eqs 51 and 53 we obtain

$$\begin{array}{c} C\left(u\right)\approx \frac{W_{\text{tot}}L^{2}r_{\text{av}}^{2}}{4\pi \sigma^{2}}\int_{0}^{\infty }\;d\tau \;K\left(\tau \right)\;\exp\;(-\frac{{\left|u\right|}^{2}+{\left(\tau v\right)}^{2}}{4\sigma^{2}})I_{0}(-\frac{|u|\tau v}{2\sigma^{2}}) \end{array}$$ (54)

where $I_{0}\left(x\right)≔1/\left(2\pi \right)\int_{0}^{2\pi }d\varphi \;\exp\left(x\cos\left(\varphi \right)\right)$ is the zeroth-order modified Bessel function of the first kind.

#### Fourier spectrum of the input correlation function

Here we compute the Fourier spectrum of the correlation function *C* in Eq 51. First, we observe that the second integral in Eq 51 is a two-dimensional cross-correlation in the variable **z** between the functions *δ*(|**z**| − *τv*) and $\Psi_{0}^{\text{in}}⋆\Psi_{0}^{\text{in}}{|}_{z}$ evaluated at point **u**. Therefore, by taking the two-dimensional Fourier transform with respect to **u** at both sides of Eq 51 yields

$$\begin{array}{c} \hat{C}\left(k\right)\approx \frac{W_{\text{tot}}}{L^{2}}\;{\left|{\hat{\Psi }}_{0}^{\text{in}}\left(k\right)\right|}^{2}\int_{0}^{\infty }\;d\tau \;K\left(\tau \right)\;\underset{=J_{0}\left(\tau v|k|\right)}{\underset{︸}{\int dz\;\frac{\delta (|z|-\tau v)}{2\pi \tau v}\;\;\exp\left(2\pi j\;z\cdot k\right)}}\; \end{array}$$ (55)

where we defined the Fourier transform pair:

$$\begin{array}{c} \hat{C}\left(k\right)≔\int \;du\;C\left(u\right)\exp\left(-2\pi j\;k\cdot u\right)\;;\;C\left(u\right)=\frac{1}{{\left(2\pi \right)}^{2}}\int \;dk\;\hat{C}\left(k\right)\exp\;\left(2\pi j\;k\cdot u\right)\; \end{array}$$ (56)

with **k** ⋅ **u** = |**k**||**u**| cos(*θ*), and we used the definition of the zeroth-order Bessel function

$$\begin{array}{c} J_{0}\left(k\right)≔\frac{1}{2\pi }\int_{0}^{2\pi }d\theta \exp\left(2\pi jk\;\cos\left(\theta \right)\right)\;. \end{array}$$ (57)

Because the tuning function $\Psi_{0}^{\text{in}}\left(x\right)≔G\left(|x|\right)$ is circularly symmetric, its two-dimensional Fourier transform ${\hat{\Psi }}_{0}^{\text{in}}\left(k\right)$ is proportional to the zeroth-order Hankel transform of G:

$$\begin{array}{c} {\hat{\Psi }}_{0}^{\text{in}}\left(k\right)=2\pi \;\tilde{G}\left(k\right)\;\;\text{with}\;k≔\left|k\right|\;, \end{array}$$ (58)

where we defined the zeroth-order Hankel transform pair:

$$\begin{array}{c} \tilde{G}\left(k\right)≔\int_{0}^{\infty }\;dr\;r\;G\left(r\right)J_{0}\left(kr\right)\;\text{and}\;G\left(r\right)=\int_{0}^{\infty }\;dk\;k\;\tilde{G}\left(k\right)J_{0}\left(kr\right)\;. \end{array}$$ (59)

By using Eq 58 in Eq 55 we obtain

$$\begin{array}{c} \hat{C}\left(k\right)=W_{\text{tot}}\frac{4\pi^{2}}{L^{2}}{\tilde{G}}^{2}\left(k\right)\int_{0}^{\infty }\;d\tau \;K\left(\tau \right)\;J_{0}\left(\tau vk\right) \end{array}$$ (60)

and by defining the equivalent adaptation kernel in space

$$\begin{array}{c} K_{\text{sp}}\left(r\right)≔\frac{1}{rv}K\;(\frac{r}{v}) \end{array}$$ (61)

we find

$$\begin{array}{c} \hat{C}\left(k\right)=W_{\text{tot}}\frac{4\pi^{2}}{L^{2}}{\tilde{G}}^{2}\left(k\right){\tilde{K}}_{\text{sp}}\left(k\right)\;. \end{array}$$ (62)

Finally, the zeroth-order Hankel transforms of the Gaussian tuning curve G (Eq 9) and of the adaptation kernel in space *K*_sp_ (Eqs 61 and 2) read

$$\begin{array}{ccc} \tilde{G}\left(k\right) & = & \frac{L^{2}r_{\text{av}}}{2\pi }\exp\;(-\frac{k^{2}\sigma^{2}}{2}) \end{array}$$ (63)

$${\tilde{K}}_{\text{sp}}\left(k\right)=\frac{1}{\tau_{\text{S}}v}{\left[k^{2}+{\left(\tau_{\text{S}}v\right)}^{-2}\right]}^{-0.5}-\frac{\mu }{\tau_{\text{L}}v}{\left[k^{2}+{\left(\tau_{\text{L}}v\right)}^{-2}\right]}^{-0.5}.$$ (64)

### Eigenvalue spectrum for spatially-irregular inputs

In this section we estimate the expected eigenvalue spectrum 〈λ_irr_(**k**)〉 for spatially-irregular inputs (Secs Spatially-irregular inputs and Pattern formation with spatially-irregular inputs). We recall that, for spatially-regular inputs, in Sec Mathematical results on grid-pattern formation we obtained (Eq 32):

$$\begin{array}{c} \lambda \left(k\right)\approx \rho \frac{W_{\text{tot}}}{L^{2}}\;\underset{\overset{\left(58\right)}{=}\;{\left|{\hat{\Psi }}_{0}^{\text{in}}\left(k\right)\right|}^{2}}{\underset{︸}{4\pi^{2}{\tilde{G}}^{2}\left(k\right)}}{\tilde{K}}_{\text{sp}}\left(k\right)-a\;\text{with}\;k≔\left|k\right|\neq 0 \end{array}$$ (65)

where $\tilde{G}$ and ${\tilde{K}}_{\text{sp}}$ are the zeroth-order Hankel transforms of the input tuning curve G (Eq 9) and of the equivalent adaptation kernel in space *K*_sp_ (Eqs 31 and 61). Note that the parameters *ρ*, *L*, *W*_tot_, and *a* do not depend on *k*. From Eq 65, the eigenvalue spectrum λ(**k**) is linearly-related to the input power spectrum $|{\hat{\Psi }}_{0}^{\text{in}}{\left(k\right)|}^{2}$ where $\Psi_{0}^{\text{in}}\left(x\right)≔G\left(|x|\right)$ is an input tuning curve centered at the origin **0** ≔ (0, 0) (Sec Input correlation for spatially-regular inputs).

Here, in analogy to Eq 65, we assume that the expected eigenvalue spectrum 〈λ_irr_(**k**)〉 for spatially-irregular inputs is linearly-related to the expected input power $\langle |{\hat{\Psi }}_{p}^{\text{in}}{\left(k\right)|}^{2}\rangle$, that is,

$$\begin{array}{c} \langle \lambda_{\text{irr}}\left(k\right)\rangle \approx \rho \frac{W_{\text{tot}}}{L^{2}}\;\langle |{\hat{\Psi }}_{p}^{\text{in}}{\left(k\right)|}^{2}\rangle {\tilde{K}}_{\text{sp}}\left(k\right)-a\;\text{with}\;k\neq 0 \end{array}$$ (66)

where ${\hat{\Psi }}_{p}^{\text{in}}\left(k\right)$ is the two-dimensional Fourier transform of the spatially-irregular tuning curve $\Psi_{p}^{\text{in}}\left(x\right)$, and the angular brackets denote statistical expectation across input realizations (see Eq 56 for a definition of the two-dimensional Fourier transform). The validity of this assumption is confirmed numerically at the end of this section.

Let us compute the expected input power spectrum $\langle |{\hat{\Psi }}_{p}^{\text{in}}{\left(k\right)|}^{2}\rangle$. We recall that the input maps $\Psi_{p}^{\text{in}}\left(x\right)$ are obtained by the superimposing *M* Gaussian receptive fields (Eq 10)

$$\begin{array}{c} \Psi_{p}^{\text{in}}\left(x\right)≔\frac{1}{\beta_{p}}\;\sum_{m=1}^{M}\;A_{pm}\;G(|x-r_{pm}|)\;\text{for}\;p=1,2,\ldots ,N \end{array}$$ (67)

with

$$\begin{array}{c} G\left(r\right)\;\overset{\left(9\right)}{=}\;\frac{L^{2}r_{\text{av}}}{2\pi \sigma^{2}}\exp\;(-\frac{r^{2}}{2\sigma^{2}})\;\text{and}\;\beta_{p}≔\sum_{m=1}^{M}\;A_{pm}\;. \end{array}$$ (68)

The field amplitudes *A*_*pm*_ ≥ 0 are uniformly distributed in the range (0, 1), and the receptive field centers **r**_*pm*_ are uniformly distributed in the environment (see Fig 9A for examples). From Eq 67 we derive

$$|{\hat{\Psi }}_{p}^{\text{in}}\left(k\right)|=\frac{2\pi }{\beta_{p}}\tilde{G}\left(k\right)\underset{≕\;\alpha_{p}}{\underset{︸}{\left|\overset{M}{\underset{m=1}{\sum }}A_{pm}\;\text{exp}\left(-2\pi j\;r_{pm}\cdot k\right)\right|}}$$ (69)

where $\tilde{G}\left(k\right)$ is the zeroth-order Hankel transform of the Gaussian function G(r). In deriving Eq 69, we used the shift property of the Fourier transform and the equivalence between the Fourier and the zeroth-order Hankel transforms for circularly-symmetric functions (Eq 58). Finally, from Eq 69 we obtain

$$\begin{array}{c} \langle |{\hat{\Psi }}_{p}^{\text{in}}{\left(k\right)|}^{2}\rangle =4\pi^{2}{\tilde{G}}^{2}\left(k\right)\Phi \;\text{with}\;\Phi ≔\langle \frac{\alpha_{p}^{2}}{\beta_{p}^{2}}\rangle \;. \end{array}$$ (70)

Therefore, for spatially-irregular inputs, the expected power spectrum $\langle |{\hat{\Psi }}_{p}^{\text{in}}{\left(k\right)|}^{2}\rangle$ is proportional to the power spectrum $4\pi^{2}{\tilde{G}}^{2}\left(k\right)$ of a single Gaussian G with scale factor Φ ≥ 0. Note that for |**k**| = 0 we obtain Φ = 1 (Eqs 69 and 70), which means that the average rate *r*_av_ is independent of the number *M* of input receptive fields and their specific spatial arrangement. Using Eq 70 in Eq 66 yields

$$\begin{array}{c} \langle \lambda_{\text{irr}}\left(k\right)\rangle \approx \rho \frac{W_{\text{tot}}}{L^{2}}\;4\pi^{2}{\tilde{G}}^{2}\left(k\right){\tilde{K}}_{\text{sp}}\left(k\right)\Phi -a\;\text{with}\;k\neq 0\;. \end{array}$$ (71)

Finally, from Eqs 65 and 71 we find (Eq 33)

$$\begin{array}{c} \langle \lambda_{\text{irr}}\left(k\right)\rangle \approx \Phi \lambda \left(k\right)+a\left(1-\Phi \right)\;. \end{array}$$ (72)

In the next section we estimate the scale factor Φ for |**k**| > 0.

#### Approximation of the scale factor Φ

The scale factor

$$\begin{array}{c} \Phi \overset{\left(70\right)}{≔}\langle \frac{\alpha_{p}^{2}}{\beta_{p}^{2}}\rangle \end{array}$$ (73)

is the second moment of the ratio of the random variables

$$\begin{array}{c} \alpha_{p}\overset{\left(69\right)}{≔}|\sum_{m=1}^{M}\;A_{pm}\;\exp\left(-2\pi j\;r_{pm}\cdot k\right)|\;\text{and}\;\beta_{p}\overset{\left(68\right)}{≔}\sum_{m=1}^{M}\;A_{pm} \end{array}$$ (74)

where the field amplitudes *A*_*pm*_ ≥ 0 are independently and uniformly distributed in the range (0, 1) and the field centers **r**_*pm*_ are independently and uniformly distributed in a square of side-length *L*.

In general, for two random variables *x* and *y*, the first order Taylor expansion of the ratio *f*(**z**) = *x*/*y* around the expected value ***μ*** ≔ (〈*x*〉, 〈*y*〉) is

$$\begin{array}{c} f\left(z\right)=f\left(\mu \right)+f_{x}\left(\mu \right)\Delta_{x}+f_{y}\left(\mu \right)\Delta_{y}+o\left(\Delta_{x}^{2}\right)+o\left(\Delta_{y}^{2}\right)+o\left(\Delta_{x}\Delta_{y}\right) \end{array}$$ (75)

where **z** ≔(*x*, *y*), Δ_*x*_ ≔ *x* − 〈*x*〉, Δ_*y*_ ≔ *y* − 〈*y*〉, and *f*_*x*_ and *f*_*y*_ are the derivatives of *f* with respect to *x* and *y*. Therefore

$$\begin{array}{c} \mathrm{Var}(\frac{x}{y})=\langle {\left[f\left(z\right)-f\left(\mu \right)\right]}^{2}\rangle =f_{x}^{2}\left(\mu \right)\mathrm{Var}\left(x\right)+f_{y}^{2}\left(\mu \right)\mathrm{Var}\left(y\right)+\\ 2f_{x}\left(\mu \right)f_{y}\left(\mu \right)\mathrm{Cov}\left(x,y\right)+\sum_{k=0}^{4}\;o\left(\langle \Delta_{x}^{k}\Delta_{y}^{4-k}\rangle \right)\;. \end{array}$$ (76)

By neglecting the higher-order joint moments $\sum_{k=0}^{4}o\left(\langle \Delta_{x}^{k}\Delta_{y}^{4-k}\rangle \right)$ and substituting *f*_*x*_(***μ***) = 1/〈*y*〉 and *f*_*y*_(***μ***) = −〈*x*〉/〈*y*〉^2^ we obtain

$$\begin{array}{c} \mathrm{Var}(\frac{x}{y})\approx \frac{{\langle x\rangle }^{2}}{{\langle y\rangle }^{2}}[\frac{\mathrm{Var}(x)}{{\langle x\rangle }^{2}}+\frac{\mathrm{Var}(y)}{{\langle y\rangle }^{2}}-2\frac{\mathrm{Cov}(x,y)}{\langle x\rangle \langle y\rangle }] \end{array}$$ (77)

and

$$\begin{array}{c} \langle \frac{x^{2}}{y^{2}}\rangle \approx \frac{{\langle x\rangle }^{2}}{{\langle y\rangle }^{2}}[\frac{\langle x^{2}\rangle }{{\langle x\rangle }^{2}}+\frac{\langle y^{2}\rangle }{{\langle y\rangle }^{2}}-2\frac{\mathrm{Cov}(x,y)}{\langle x\rangle \langle y\rangle }-1]\;. \end{array}$$ (78)

In the following, we use Eq 78 to approximate the scale factor Φ (Eq 73).

We start by giving an intuitive interpretation of the random variables *α*_*p*_ and *β*_*p*_. Consider a *M*-steps random walk on the complex plane with random directions **r**_*pm*_ ⋅ **k** and random step sizes *A*_*pm*_. The coefficients *α*_*p*_ measure the total distance traveled by the random walker, and the coefficients *β*_*p*_ measure the total length of the path (Eq 74). Note that the larger the number of steps *M*, the smaller is the correlation between the distance traveled *α*_*p*_ and the total path length *β*_*p*_, i.e., |Cov(*α*_*p*_, *β*_*p*_)| ≪ 1 for *M* ≫ 1. In this case we can neglect the covariance term in Eq 78, and the factor Φ is approximated by knowing only the first two moments of the distributions of *α*_*p*_ and *β*_*p*_.

For |**k**| > 1/*L*, the random directions **r**_*pm*_ ⋅ **k** (mod 1) are approximately uniformly distributed in the range (0, 1). In this case, the traveled distance *α*_*p*_ follows a Rayleigh distribution with density [149]

$$\begin{array}{c} f\left(\alpha_{p}\right)=\frac{2\alpha_{p}}{M\langle A_{pm}^{2}\rangle }\exp\;(-\frac{\alpha_{p}^{2}}{M\langle A_{pm}^{2}\rangle }) \end{array}$$ (79)

where $\langle A_{pm}^{2}\rangle =1/3$ for *A*_*pm*_ uniformly distributed in interval (0, 1). Therefore, the first two moments of *α*_*p*_ read

$$\begin{array}{c} \langle \alpha_{p}\rangle =\sqrt{\frac{M\pi }{12}}\;\text{and}\;\langle \alpha_{p}^{2}\rangle =\frac{M}{3}\;. \end{array}$$ (80)

The total path length *β*_*p*_ is the sum of *M* random variables uniformly distributed in (0, 1), which follows an Irwin-Hall distribution. Therefore, the first two moments of *β*_*p*_ are

$$\begin{array}{c} \langle \beta_{p}\rangle =\frac{M}{2}\;\text{and}\;\langle \beta_{p}^{2}\rangle =\frac{M+3M^{2}}{12}\;. \end{array}$$ (81)

Finally, by using Eqs 80 and 81 in Eq 73 we obtain

$$\begin{array}{ccc} \Phi (M)\approx & \frac{\pi }{3M}\;(\frac{4}{\pi }+\frac{1}{3M})\; & \text{for}\;M>1\;\text{and}\;|k|>1/L \end{array}$$ (82)

$$\begin{array}{ccc} \approx & \frac{4}{3M}\; & \text{for}\;M>3\;\text{and}\;|k|>1/L\;. \end{array}$$ (83)

**Fig 10:**
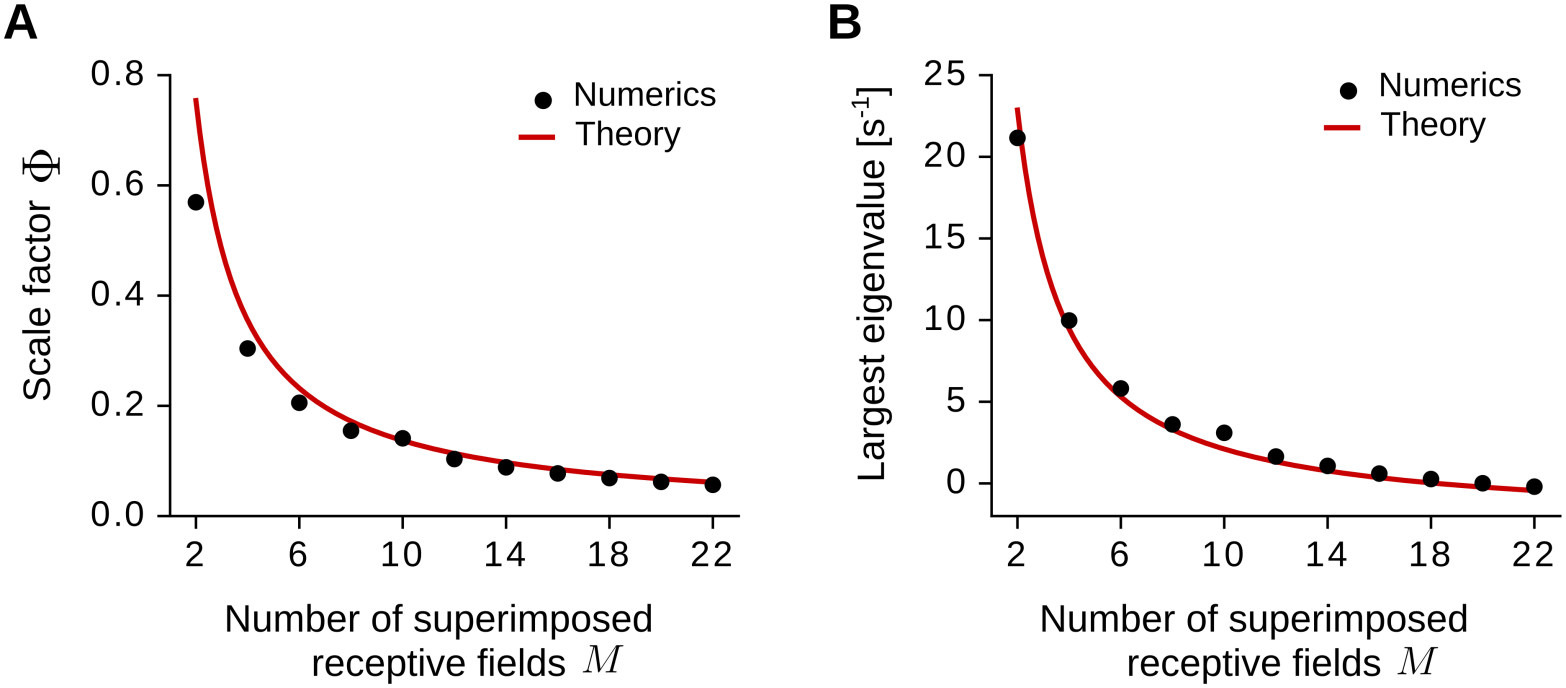
Scale factor Φ and largest eigenvalue λ_max_ for spatially-irregular inputs. (A) The scale factor Φ for *M* > 1 superimposed fields (Eq 70). The black dots are obtained by estimating the power spectrum at frequency |**k**| = 1 m^−1^ for 3600 input realizations. The red line is the theoretical curve in Eq 82. (B) The largest eigenvalue λ_max_ as a function of the number of superimposed fields *M*. The black dots are obtained by computing the eigenvalues of the correlation matrix *C_ij_* − *aδ_ij_* for *N* = 3600 inputs, where *δ_ij_* is the Kronecker delta (Eq 21). The red line is obtained from Eqs 71 and 82. Note that, according to Eq 71, the largest eigenvalue is always at the critical frequency *k*_max_ = 3 m^−1^ for any value of *M*. Parameter values as in Fig 9 (see Sec Numerical simulations).

Fig 10A shows the scale factor Φ as a function of the number *M* of superimposed Gaussian fields (Eq 82). Note that the approximation is more accurate for large values of *M*, which correspond to lower values of |Cov(*α*_*p*_, *β*_*p*_)|. Fig 10B shows the largest eigenvalue in the system as a function of *M*. The good match between the theoretical curve and the numerical estimations supports the validity of Eq 66. Additionally, Eq 66 predicts that, irrespectively of the value of *M*, the largest eigenvalue λ_max_ = λ(*k*_max_) is always at the critical frequency of *k*_max_ = 3 m^−1^ for *σ* = 6.25 cm and *τ*_L_ = 0.16 s, which matches the numerical results in Fig 9.

### Pattern formation with after-spike potentials

Here we study whether grid-like patterns could emerge by means of after-spike hyperpolarizing potentials (see discussion in Sec Spike-rate adaptation). To this end, we consider a model of the output neural activity that is alternative to the one presented in the main text (Sec Model of neural activity, Eq 1). We model input post-synaptic potentials (PSPs) with a kernel *K*^in^ applied to the input spike trains $S_{j}^{\text{in}}$, and we model output after-spike hyperpolarizing potentials (AHPs) with a kernel *K*^out^ applied to the output spike train *S*^out^:

$$\begin{array}{c} r^{\text{out}}\left(t\right)≔r_{0}+\int_{0}^{\infty }\;\;ds\;K^{\text{out}}\left(s\right)S^{\text{out}}\left(t-s\right)+\int_{0}^{\infty }\;\;d\tau \;K^{\text{in}}\left(\tau \right)\sum_{j=1}^{N}\;w_{j}S_{j}^{\text{in}}\left(t-\tau \right)\; \end{array}$$ (84)

where *r*_0_ ≥ 0 is a baseline firing rate.

First, we show that the average dynamics of Eq 84 can be rewritten in terms of an equivalent kernel *K*^eq^ applied to the input spikes only. We average Eq 84 across input and output spike train realizations:

$$\begin{array}{c} \langle r^{\text{out}}\left(t\right)\rangle =r_{0}+\int_{0}^{\infty }\;\;ds\;K^{\text{out}}\left(s\right)\langle r^{\text{out}}\left(t-s\right)\rangle +\int_{0}^{\infty }\;\;d\tau \;K^{\text{in}}\left(\tau \right)\sum_{j=1}^{N}\;w_{j}r_{j}^{\text{in}}\left(t-\tau \right)\;. \end{array}$$ (85)

And by taking the Fourier transform

$$\begin{array}{c} \hat{f}\left(\omega \right)≔\int \;dt\;f\left(t\right)\;\exp\left(-j\omega t\right)\;;\;f\left(t\right)=\frac{1}{2\pi }\int \;d\omega \;\hat{f}\left(\omega \right)\;\exp\left(j\omega t\right)\; \end{array}$$ (86)

at both sides of Eq 85 we obtain

$$\begin{array}{c} \langle {\hat{r}}^{\text{out}}\left(\omega \right)\rangle =r_{0}\delta \left(\omega \right)+\frac{{\hat{K}}^{\text{in}}\left(\omega \right)}{1-{\hat{K}}^{\text{out}}\left(\omega \right)}\sum_{j=1}^{N}\;w_{j}{\hat{r}}_{j}^{\text{in}}\left(\omega \right)\;. \end{array}$$ (87)

From Eqs 85 to 87 we assumed that the input and the output kernels are causal, i.e., *K*^in,out^(*t*) = 0 for *t* < 0, and that the output kernel has integral different from 1, i.e., ${\hat{K}}^{\text{out}}\left(0\right)=\int_{0}^{\infty }dt\;K^{\text{out}}\left(t\right)\neq 1$. Finally, by defining the equivalent filter

$$\begin{array}{c} {\hat{K}}^{\text{eq}}\left(\omega \right)≔\frac{{\hat{K}}^{\text{in}}\left(\omega \right)}{1-{\hat{K}}^{\text{out}}\left(\omega \right)}\;, \end{array}$$ (88)

the inverse Fourier transform of Eq 87 reads

$$\begin{array}{c} \langle r^{\text{out}}\left(t\right)\rangle =r_{0}+\int_{0}^{\infty }\;\;d\tau \;K^{\text{eq}}\left(\tau \right)\sum_{j=1}^{N}\;w_{j}r_{j}^{\text{in}}\left(t-\tau \right)\;, \end{array}$$ (89)

which is equivalent to Eq 15 with *K*^eq^ = *K*.

Next, we compute the equivalent filter *K*^eq^ for a simple choice of the input and output kernels

$$\begin{array}{c} K^{\text{in}}\left(t\right)≔\{\begin{array}{cc} \frac{1}{\tau_{\text{in}}}\;\exp\;(-\frac{t}{\tau_{\text{in}}}) & \text{for}\;t\geq 0\\ 0 & \text{for}\;t<0 \end{array} \end{array}$$ (90)

and

$$\begin{array}{c} K^{\text{out}}\left(t\right)≔\{\begin{array}{cc} -\frac{\mu_{\text{out}}}{\tau_{\text{out}}}\;\exp\;(-\frac{t}{\tau_{\text{out}}}) & \text{for}\;t\geq 0\\ 0 & \text{for}\;t<0 \end{array} \end{array}$$ (91)

where *τ*_in_, *τ*_out_ > 0 are decay time constants, and the parameter *μ*_out_ > 0 scales the integral of the output kernel $\int_{0}^{\infty }dt\;K^{\text{out}}\left(t\right)=-\mu_{\text{out}}$. We assume that the input kernel *K*^in^ (modeling an incoming PSP) decays faster than the output kernel *K*^out^ (modeling an output AHP), i.e., *τ*_in_ < *τ*_out_. From the definition of the filter *K*^eq^ in Eq 88 we obtain

$$\begin{array}{c} {\hat{K}}^{\text{eq}}\left(\omega \right)=\underset{≕H}{\underset{︸}{\frac{1/\tau_{\text{in}}}{1/\tau_{\text{in}}-\left(1+\mu_{\text{out}}\right)/\tau_{\text{out}}}}}[\frac{1/\tau_{\text{in}}-1/\tau_{\text{out}}}{1/\tau_{\text{in}}+j\omega }-\frac{\mu_{\text{out}}/\tau_{\text{out}}}{\left(1+\mu_{\text{out}}\right)/\tau_{\text{out}}+j\omega }] \end{array}$$ (92)

where we used

$$\begin{array}{c} {\hat{K}}^{\text{in}}\left(\omega \right)=\frac{1/\tau_{\text{in}}}{1/\tau_{\text{in}}+j\omega }\;\text{and}\;{\hat{K}}^{\text{out}}\left(\omega \right)=-\frac{\mu_{\text{out}}/\tau_{\text{out}}}{1/\tau_{\text{out}}+j\omega }\;. \end{array}$$ (93)

Finally, the inverse Fourier transform of Eq 92 reads

$$\begin{array}{c} K^{\text{eq}}\left(t\right)=H\cdot [(\frac{1}{\tau_{\text{in}}}-\frac{1}{\tau_{\text{out}}})\exp\;(-\frac{t}{\tau_{\text{in}}})-\frac{\mu_{\text{out}}}{\tau_{\text{out}}}\exp\;(-\frac{t}{\tau_{\text{out}}/\left(1+\mu_{\text{out}}\right)})] \end{array}$$ (94)

for *t* ≥ 0 and *K*^eq^(*t*) = 0 for *t* < 0. Eq 94 shows that the equivalent filter *K*^eq^ is a difference of two exponentials, similarly to the kernel *K* in Eq 2. Note however that the two exponentials are scaled differently as compared to the original filter *K*. Additionally, if the integral of the output kernel is negative, the integral of the equivalent filter is always positive (Eq 88 with *ω* = 0).

**Fig 11:**
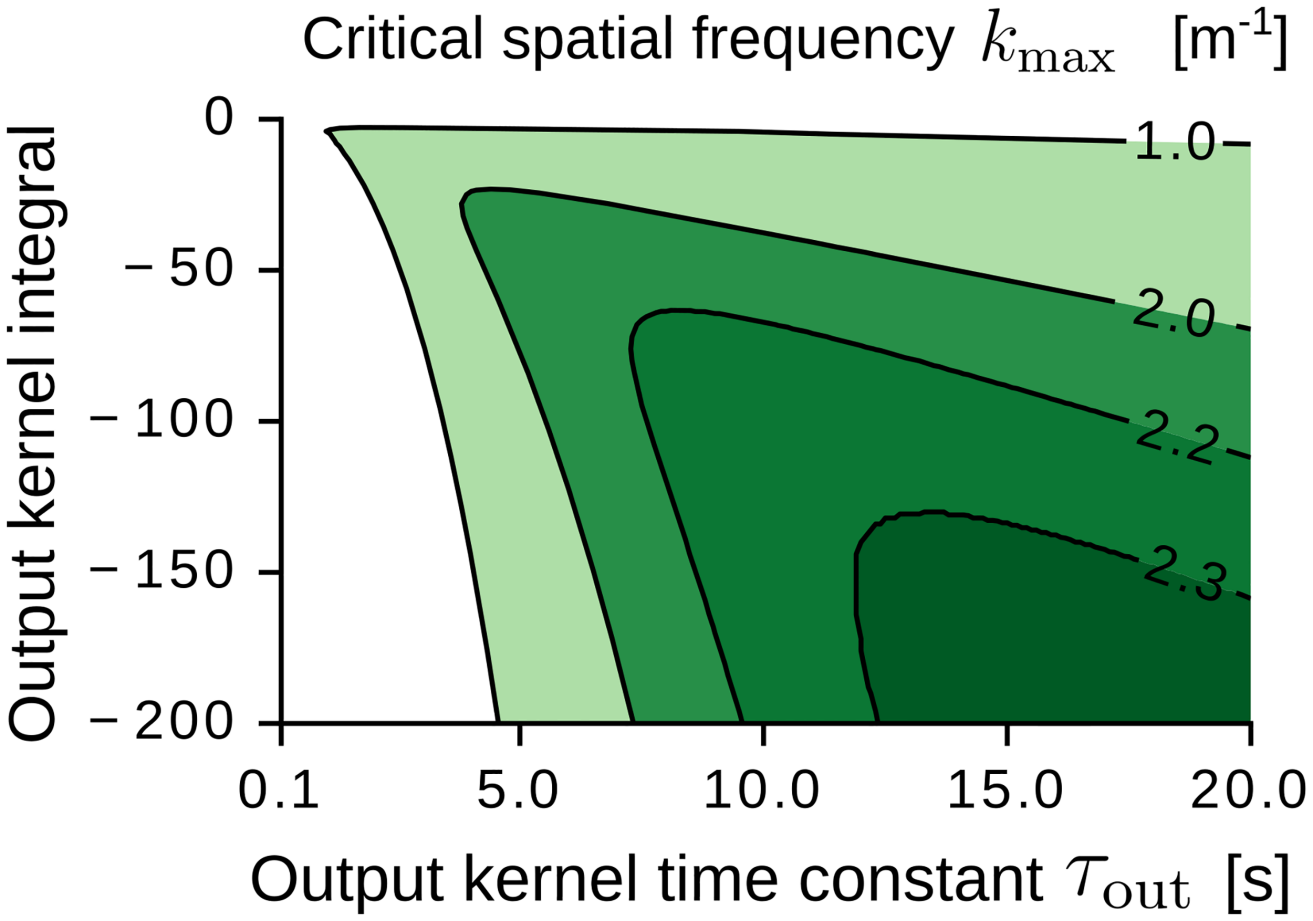
Grid scale with after-spike hyperpolarizing potentials. The critical spatial frequency *k*_max_ is plotted as a function of the output-kernel integral −*μ*_out_ and the output-kernel time constant *τ*_out_ (Eqs 31 and 32 with *K* = *K*^eq^). The black lines are iso-levels (see annotated values). Regions enclosed by two adjacent iso-lines are colored uniformly (darker colors denote larger values). The input-kernel time constant is *τ*_in_ = 5 ms. Similar results are obtained with different values of *τ*_in_ < *τ*_out_. Parameter values: *σ* = 6.25 cm, *v* = 0.25 m/s, *L* = 1 m. *r*_av_ = 0.4 s^−1^.

To test whether spatially-periodic patterns could still emerge in this scenario, we compute the eigenvalue spectrum λ(*k*) and the critical spatial frequency *k*_max_ by using Eqs 31 and 32 with *K* = *K*^eq^. Surprisingly, we find that typical grid scales (e.g., *k*_max_ > 2 m^−1^) are obtained for output-kernel time constants of the order of seconds, which seem biologically unrealistic (Fig 11). Therefore, we conclude that AHPs alone are not sufficient to generate grid-like patterns. Nevertheless, AHPs could still support structure formation by amplifying the effects of a band-pass filter that is already present at the input.

### Numerical simulations

Model parameters and derived quantities are summarized in Tables 1 and 2.

**Table 1:** Model parameters.

Neural activity		
*N*		Number of synaptic inputs
*r*_0_	[s^−1^]	Baseline rate of the output neuron
*τ*_S_	[s]	Adaptation kernel short time constant
*τ*_L_	[s]	Adaptation kernel long time constant
*μ*		Adaptation kernel scaling parameter
Spatial exploration		
*L*	[m]	Side-length of the arena
*v*	[m/s]	Running speed of the virtual rat
*σ*_*θ*_		Standard deviation of running directions
Input spatial tuning		
*r*_av_	[s^−1^]	Average input rate in the arena
*σ*	[m]	Width of the input receptive fields
*M*		Number of receptive fields per neuron (spatially-irregular inputs)
Synaptic plasticity		
*η*		Learning rate
*τ*_W_	[s]	Decay time constant of the learning window *W*
*W*_tot_	[s]	Integral of the learning window *W*
*α*		Multiplicative weight-normalization constant
*β*		Additive weight-normalization constant
Derived quantities		
*a*	[s^−1^]	Multiplicative weight-normalization rate
*b*	[s^−1^]	Additive weight-normalization rate
λ_max_	[s^−1^]	Maximal eigenvalue
$w_{\text{av}}^{\infty }$		Average synaptic weight
*τ*_av_	[s]	Weight normalization time scale
*τ*_str_	[s]	Structure formation time scale

**Table 2:** Default parameter values for the numerical simulations. See also Table 1 for short descriptions of the parameters. TL: top-left, TR: top-right, BL: bottom-left, BR: bottom-right. Note that in Fig 6A the learning rate *η* is varied from 2 ⋅ 10^−5^ to 10 ⋅ 10^−5^ and that in Fig 6B the virtual-rat running speed is sampled from an Ornstein-Uhlenbeck process with long-term mean $\bar{v}=v$.

	Unit	Fig 5 Fig 6	Fig 7 (TL)	Fig 7 (TR)	Fig 7 (BL) Fig 8A1–8A4	Fig 7 (BR) Fig 8B1–8B4	Fig 9 Fig 10
*N*		900	3600	3600	3600	3600	3600
*r*_0_	[s^−1^]	10	4	4	4	4	4
*τ*_S_	[s]	0.1	0.1	0.1	0.1	0.1	0.1
*τ*_L_	[s]	0.16	0.16	0.35	0.16	0.35	0.16
*μ*		1.06	1.06	1.06	1.06	1.06	1.06
*L*	[m]	1	2	2	2	2	1
*v*	[m/s]	0.25	0.25	0.25	0.25	0.25	0.25
*σ*_*θ*_		0.7	0.7	0.7	0.7	0.7	0.7
*r*_av_	[s^−1^]	0.4	0.21	0.085	0.3	0.1	0.8
*σ*	[m]	0.0625	0.045	0.045	0.0625	0.0625	0.0625
*M*		−	−	−	−	−	10
*η*		2 ⋅ 10^−5^	5 ⋅ 10^−5^	5 ⋅ 10^−5^	5 ⋅ 10^−5^	5 ⋅ 10^−5^	5 ⋅ 10^−5^
*τ*_W_	[s]	0.05	−	−	−	−	−
*W*_tot_	[s]	1	1	1	1	1	1
*α*		3.56	−	−	−	−	−
*β*		−8.78	−	−	−	−	−
*a*	[s^−1^]	1.1	4	4	4	4	2.5
*b*	[s^−1^]	0.49	0.69	0.28	1.23	0.31	2.8
λ_max_	[s^−1^]	1	0.90	0.82	0.80	0.85	1.75
$w_{\text{av}}^{\infty }$		0.05	0.05	0.05	0.05	0.05	0.02
*τ*_av_	[s]	5.13 ⋅ 10^3^	1.44 ⋅ 10^3^	3.57 ⋅ 10^3^	8.12 ⋅ 10^2^	3.18 ⋅ 10^3^	1.42 ⋅ 10^2^
*τ*_str_	[s]	5 ⋅ 10^4^	2.23 ⋅ 10^4^	2.42 ⋅ 10^4^	2.50 ⋅ 10^4^	2.36 ⋅ 10^4^	1.14 ⋅ 10^4^

#### Simulation of the detailed spiking model

The detailed spiking model (Figs 5 and 6) is simulated using the Brian2 simulation software [150]. Neural and synaptic variables are integrated with a time step of 1 ms. The random walk of the virtual rat that is updated every 10 ms. The physical space explored by the virtual rat is discretized in 200^2^ square bins.

#### Simulation of the averaged weight dynamics

The average weight dynamics (Eq 16) is integrated by using the forward Euler method with integration time step of 50 s (Figs 7–9). The input correlation matrix *C* is computed using Eq 54 for spatially-regular inputs, and using Eq 21 for spatially-irregular inputs.

#### Initialization of the synaptic weights

At the initial condition the synaptic weights are normally distributed around the target normalization level $w_{\text{av}}^{\infty }=5\cdot 10^{-3}$. The standard deviation is 10^−4^ for the spiking simulations and 10^−3^ for the average weight dynamics.

### Data analysis

#### Grid properties

We compute the grid spatial scale from the two-dimensional Fourier amplitude of the grid pattern. We estimate the radial amplitude profile by averaging over the angular dimension. We then define the grid scale as the frequency where the amplitude profile has a global maximum.

The grid orientation is estimated from the spatial auto-correlogram of the grid pattern. We detect the peak closest to the center in the first quadrant of the auto-correlogram. We then define the grid orientation as the angle between the detected peak and the horizontal axis.

We define the grid spatial phase as the position of the closest peak to the center in the cross-correlation between the grid pattern and a reference grid at the same scale.

#### Gridness score

We estimate the gridness score similarly to Langston et al. [135]. First, we compute the spatial auto-correlogram of the weight (or firing-rate) pattern and we retain only points within a ring of outer radius *R*_*i*_ and inner radius *R*_*i*_/2. We then compute the gridness score *g*_*i*_ as

$$\begin{array}{c} g_{i}≔\frac{1}{2}[\rho_{i}\left(60\right)+\rho_{i}\left(120\right)]-\frac{1}{3}[\rho_{i}\left(30\right)+\rho_{i}\left(90\right)+\rho_{i}\left(150\right)] \end{array}$$ (95)

where *ρ*_*i*_(*φ*) is the Pearson’s correlation coefficient between the original ring (of outer radius *R*_*i*_) and the same ring rotated by *φ* degrees. The final gridness score is defined as the maximum *g*_*i*_ by varying the outer radius *R*_*i*_ between 0.7/*k*_max_ and 2.5/*k*_max_ where *k*_max_ is the spatial frequency of the pattern.

#### Estimation of output firing-rate maps

The output firing-rate maps Ψ^out^ in Fig 9B are computed as follows:

$$\begin{array}{c} \Psi^{\text{out}}\left(x\right)=r_{0}+\int dy\;K_{\text{sp}}\left(|y|\right)\sum_{i=1}^{N}\;w_{i}\Psi_{i}^{\text{in}}\left(x-y\right) \end{array}$$ (96)

where *r*_0_ is the baseline firing rate, *w*_*i*_ are the synaptic weights at the end of the simulation, $\Psi_{i}^{\text{in}}$ are the input spatial maps, and *K*_sp_ is the equivalent adaptation kernel in space (Eq 31). The convolution with the filter *K*_sp_ accounts for the average effect of the temporal kernel *K* on the output firing rate.
